# Reciprocal interaction between I_K1_ and I_f_ in biological pacemakers: A simulation study

**DOI:** 10.1371/journal.pcbi.1008177

**Published:** 2021-03-10

**Authors:** Yacong Li, Kuanquan Wang, Qince Li, Jules C. Hancox, Henggui Zhang

**Affiliations:** 1 School of Computer Science and Technology, Harbin Institute of Technology, Harbin, China; 2 Peng Cheng Laboratory, Shenzhen, China; 3 School of Physiology, Pharmacology and Neuroscience, Medical Sciences Building, University Walk, Bristol, United Kingdom; 4 Biological Physics Group, School of Physics and Astronomy, The University of Manchester, Manchester, United Kingdom; 5 Key Laboratory of Medical Electrophysiology of Ministry of Education and Medical Electrophysiological Key Laboratory of Sichuan Province, Institute of Cardiovascular Research, Southwest Medical University, Luzhou, China; University of Virginia, UNITED STATES

## Abstract

Pacemaking dysfunction (PD) may result in heart rhythm disorders, syncope or even death. Current treatment of PD using implanted electronic pacemakers has some limitations, such as finite battery life and the risk of repeated surgery. As such, the biological pacemaker has been proposed as a potential alternative to the electronic pacemaker for PD treatment. Experimentally and computationally, it has been shown that bio-engineered pacemaker cells can be generated from non-rhythmic ventricular myocytes (VMs) by knocking out genes related to the inward rectifier potassium channel current (I_K1_) or by overexpressing hyperpolarization-activated cyclic nucleotide gated channel genes responsible for the “funny” current (I_f_). However, it is unclear if a bio-engineered pacemaker based on the modification of I_K1_- and I_f_-related channels simultaneously would enhance the ability and stability of bio-engineered pacemaking action potentials. In this study, the possible mechanism(s) responsible for VMs to generate spontaneous pacemaking activity by regulating I_K1_ and I_f_ density were investigated by a computational approach. Our results showed that there was a reciprocal interaction between I_K1_ and I_f_ in ventricular pacemaker model. The effect of I_K1_ depression on generating ventricular pacemaker was mono-phasic while that of I_f_ augmentation was bi-phasic. A moderate increase of I_f_ promoted pacemaking activity but excessive increase of I_f_ resulted in a slowdown in the pacemaking rate and even an unstable pacemaking state. The dedicated interplay between I_K1_ and I_f_ in generating stable pacemaking and dysrhythmias was evaluated. Finally, a theoretical analysis in the I_K1_/I_f_ parameter space for generating pacemaking action potentials in different states was provided. In conclusion, to the best of our knowledge, this study provides a wide theoretical insight into understandings for generating stable and robust pacemaker cells from non-pacemaking VMs by the interplay of I_K1_ and I_f_, which may be helpful in designing engineered biological pacemakers for application purposes.

## Author summary

Pacemaking dysfunction has become one of the most severe cardiac diseases, which may result in arrhythmia and even death. The treatment of pacemaking dysfunction by electronic pacemakers has saved millions of people in the past fifty years. However not every patient can benefit from these because of possible limitations, such as surgical implication and lack of response to the autonomic stimulus. The development of biological pacemaker based on gene engineering technology provides a promising alternative to the electronic pacemaker by manipulating the gene expression of cardiac cells. However, it is still unclear how a stable and robust biological pacemaker can be generated in this way. The present study aims to elucidate possible mechanisms responsible for a bio-engineered pacemaker using a computational electrophysiological model by modifying ionic channel properties of I_K1_ and incorporating I_f_ in a human ventricular cell model. Simulation results indicated that the reciprocal interaction between I_K1_ and I_f_ determined the stability and ability of biological pacemaker. This study provides new insight into the understanding of the initiation of pacemaking behaviours in non-rhythmic cardiac myocytes, providing a theoretical basis for experimental design of biological pacemakers.

## Introduction

Currently, electronic pacemaker implantation is the only non-pharmacological therapy for some patients with pacemaking dysfunction. But electronic pacemakers have some possible limitations [1]. Implantation of a pacemaker device may have complications for patients, especially for aged ones because of their infirm health [2]. Pediatric patients can receive electronic pacemakers; however, the device has to be replaced as they grow, and repeated surgeries are needed [3]. Electronic devices can be subject to electromagnetic interference [4], which causes inconvenience to the patients. A further issue is that classical electronic pacemakers are insensitive not only to hormone stimulation [5] but also to autonomic emotion responsiveness [4], although there are some attempts to make them respond to autonomic nervous control [6]. In addition, the long-term use of electronic pacemakers has been reported to increase the risk of heart failure [7]. Appropriately designed biological pacemakers (bio-pacemakers) have the potential to overcome some of the limitations of electrical device use [8], such as the lack of pacing flexibility. Also, engineered bio-pacemakers could potentially involve only minor surgical trauma for implantation as well as facilitating chronotropic responses [9]. In previous experimental studies, it has been shown that a bio-pacemaker can be engineered *via* adenoviral gene transduction [10–12] or lentiviral vector [13,14] techniques, by which non-pacemaking cardiac myocytes (CMs) can be transformed to the rhythmic pacemaker-like cells.

The native cardiac primary pacemaker, sinoatrial node (SAN), is a special region comprised of cells with distinct electrophysiological properties to those of cells in the working myocardium. Such intrinsic and special electrophysiological properties of SAN cells are mainly manifested by their small if not absent inward rectifier potassium channel current (I_K1_) [15], but a large “funny” current (I_f_) [16] that is almost absent in atrial and ventricular cells. I_K1_ helps to hyperpolarize cell membrane potential and contributes to maintaining a stable negative resting potential in ventricular myocytes (VMs). But in the SAN, absence of I_K1_ makes a more positive maximum diastolic potential (MDP) than the resting potential in VMs, which helps its depolarization. I_f_ plays an important role in phase 4 depolarization of SAN action potentials, thus influences the pacemaking rate in the SAN [17]. In addition to the absence of I_K1_ and presence of I_f_, T-type Ca^2+^ channel current (I_CaT_) [18] and sustained inward current (I_st_) [19] also contribute to spontaneous pacemaking activity in SAN cells. Similar to I_f_, I_CaT_ plays a role in the depolarization in the SAN but is usually undetectable in VMs. Such unique electrophysiological properties of SAN cells formed a theoretical basis to engineer non-pacemaking CMs into spontaneous pacemaker cells. These non-pacemaker cells include native CMs, such as ventricular [11,20–22], atrial [23] or bundle branch myocytes [24]. They can also be stem cells, such as embryonic stem cells [25–27], bone marrow stem cells [13,28,29], adipose-derived stem cells [30–32], or induced pluripotent stem cell [14,33,34].

With gene therapy, these non-rhythmic cells have been manipulated to provoke automaticity. In previous studies, knocking down the *Kir2*.*1* gene to reduce the expression of I_K1_ promoted spontaneous rhythms in newborn murine VMs [20]; by reprogramming the *Kir2*.*1* gene in guinea-pigs, VMs also produced pacemaker activity when I_K1_ was suppressed [11,21]. As I_f_ plays an important role in the native SAN cell pacemaking, a parallel gene therapy manipulation to create engineered bio-pacemaker has been carried out by expressing the *HCN* gene family in non-rhythmic CMs [35]. It has been shown that expressing *HCN2* produced escape beats in canine CMs [23,24] and initiated spontaneous beats in neonatal rat VMs [22]. *HCN* expression in stem cells-induced-CMs also enhanced their pacemaking rate [13,28,29,36,37]. Overexpressing *HCN4* can also induce spontaneous pacemaking activity in mouse embryonic stem cells [38]. However, acute *HCN* gene expression might have a side effect on the normal cardiac pacemaking activity [39–41]. The overexpression of the *HCN* gene in non-rhythmic CMs can cause ectopic pacemaker automaticity and even arrhythmicity [42].

It has been suggested that a combined manipulation of I_K1_ and I_f_ may be a better alternative for creating a bio-pacemaker [43]. The expression of the transcriptional regulator *TBX18*, which influenced both I_f_ and I_K1_ expression, generated appropriate automatic responses in non-pacemaking CMs [10,32,44]. In addition, reprogramming *TBX18* in porcine VMs did not show the increase of arrhythmia risk [12], indicating the probable superiority of manipulating I_K1_ and I_f_ jointly for generating a bio-pacemaker. Furthermore, an experimental study showed that in HEK293 cells with expression of the *Kir2*.*1* and the *HCN* genes, not only I_f_ but also moderate I_K1_ was necessary to induce spontaneous rhythmic oscillations [45].

Computational modelling offers a means to investigate different approaches to generating stable pacemaking activity. Based on the cardiac cell model, bifurcation analysis was widely used to explore the effect of changes on some individual ion channel current on the pacemaking activities, such as the role of down-regulating I_K1_ in the pacemaking initiation in VMs [46–48] and the role of I_f_ in the pacemaking activity in SAN cells [49]. The interplay between multiple ion channel currents in the pacemaking initiation was also investigated in VMs [50] or SAN [51] models, with focus on their roles in sustaining the spontaneous oscillation. Up to now, the dynamic interplay between I_K1_ and I_f_ on modulating the stability of bio-pacemaker action potentials (APs) has not yet been comprehensively investigated in biophysically detailed cardiac single-cell models.

Considering the availability of experimental data on transformation of VMs into rhythmic cells [11,20–22] and the cardiac dysfunctions arising from atrioventricular heart-block, we chose the VM cell model for investigating bio-pacemaking activity. In this study, we constructed a bio-pacemaker model based on a human VMs model [52] by manipulating I_K1_ and incorporating I_f_ [53] into the model. This study aimed to investigate (i) possible mechanism(s) underlying the pacemaking activity of the VMs in the I_K1_/I_f_ parameter space; and (ii) the reciprocal interaction of reduced I_K1_ and increased I_f_ in generating stable pacemaking APs. In addition, possible factors responsible for impaired pacemaking activity due to the inappropriate ratio between I_K1_ and I_f_ were also investigated. Moreover, theoretical analysis of the role of I_CaT_ in I_K1_/I_f_ pacemaker model was investigated. This study provides insight into generating stable and robust engineered bio-pacemaker.

## Methods

### Single bio-pacemaker cell model

Previous experimental studies [10,11,20–22] implemented the suppression of *Kir2*.*1*, the incorporation of *HCN* channels and the expression of *TBX18* to induce pacemaking in VMs. In this study, we used a human VMs model built by Ten Tusscher et al. in 2006 (TP06 model) [52] as the basal model to investigate possible pacemaking mechanisms in VM-transformed pacemaking cells. In brief, the basal VM cell model can be described by the following ordinary differential equation:

$$\frac{\mathrm{dV}}{\mathrm{dt}}=–\frac{I_{\mathrm{ion}}}{C_{m}}$$ (1)

where V is the voltage across cell membrane surfaces, t is time, I_ion_ is the sum of all transmembrane ionic currents, and C_m_ cell capacitance.

The I_ion_ in the original ventricular model is described by the following equation:

$$I_{\mathrm{ion}}=I_{\mathrm{Na}}+I_{K1}+I_{\mathrm{to}}+I_{\mathrm{Kr}}+I_{\mathrm{Ks}}+I_{\mathrm{CaL}}+I_{\mathrm{NaCa}}+I_{\mathrm{NaK}}+I_{\mathrm{pCa}}+I_{\mathrm{pK}}+I_{\mathrm{bCa}}+I_{\mathrm{bNa}}$$ (2)

where I_Na_ is fast sodium channel current, I_K1_ is inward rectifier potassium channel current, I_to_ is transient outward current, I_Kr_ is rapid delayed rectifier potassium channel current, I_Ks_ is slow delayed rectifier potassium channel current, I_CaL_ is L-type calcium current, I_NaCa_ is Na^+^/Ca^2+^ exchange current, I_NaK_ is Na^+^/K^+^ pump current, I_pCa_ and I_pK_ are plateau Ca^2+^ and K^+^ currents, and I_bCa_ and I_bNa_ are background Ca^2+^ and Na^+^ currents. The formulations and their parameters for the ionic channels of human VM cells were listed in [52,54].

To mimic the reduction of *Kir2*.*1* expression [11,20,21] or the suppression of I_K1_ by expressing *TBX18* [10], in simulations I_K1_ was decreased by modulating its macroscopic channel conductance (G_K1_). To mimic the incorporation of I_f_ in VMs experimentally [22], we modified the basal model of Eq 2 by incorporating human SAN I_f_ formulation [53]. In simulations, I_f_ was modulated by changing its channel conductance (G_f_).

As a result, the I_ion_ for the bio-pacemaker model can be described as:

$$I_{\mathrm{ion}}=I_{\mathrm{Na}}+I_{K1}+I_{\mathrm{to}}+I_{\mathrm{Kr}}+I_{\mathrm{Ks}}+I_{\mathrm{CaL}}+I_{\mathrm{NaCa}}+I_{\mathrm{NaK}}+I_{\mathrm{pCa}}+I_{\mathrm{pK}}+I_{\mathrm{bCa}}+I_{\mathrm{bNa}}+I_{f}$$ (3)

where I_K1_ could be expressed by

$$I_{K1}={S_{K1}\;G}_{K1}\sqrt{\frac{K_{o}}{5.4}}x_{k1\infty }(V_{m}‐E_{k})$$ (4)

where G_K1_ is the conductance of I_K1_, x_k1∞_ is a time-independent inward rectification factor, K_o_ is extracellular K^+^ concentration and E_K_ is the equilibrium potentials of K^+^. S_K1_ is a scaling factor used to simulate the change of I_K1_ expression level.

The I_f_ channel is permeable to Na^+^ and K^+^ ions [53]. As a result, I_f_ could be described by two components:

$$I_{f}=I_{f,Na}+I_{f,K}$$ (5)

$$I_{f,Na}={S_{f}G}_{f,Na}y(V-E_{Na})$$ (6)

$$I_{f,K}=S_{f}G_{f,K}y(V-E_{K})$$ (7)

where G_f,Na_ and G_f,K_ are maximal I_f,Na_ and I_f,K_ channel conductance, y is a time-independent inward rectification factor that is a function of voltage, E_Na_, E_K_ are equilibrium potentials of Na^+^ and K^+^ channels respectively, and S_f_ is a scaling factor used to simulate the change of I_f_ expression level.

Formulations of other channel currents for the VM cell model are the same as those in the original model in [52]. The bio-pacemaker cell model can be found in S1 Code.

I_CaT_ was incorporated into the I_K1_/I_f_-induced bio-pacemaker cell model to investigate its role in generating pacemaking action potentials. Its formulations can be found in [50]. As a result, the I_ion_ is described as:

$$I_{\mathrm{ion}}=I_{\mathrm{Na}}+I_{K1}+I_{\mathrm{to}}+I_{\mathrm{Kr}}+I_{\mathrm{Ks}}+I_{\mathrm{CaL}}+I_{\mathrm{CaT}}+I_{\mathrm{NaCa}}+I_{\mathrm{NaK}}+I_{\mathrm{pCa}}+I_{\mathrm{pK}}+I_{\mathrm{bCa}}+I_{\mathrm{bNa}}+I_{f}$$ (8)

### Evaluating criterion of the pacemaking stability and ability

To analyse the effect of I_K1_ and I_f_ on pacemaking activity, we simulated the membrane potential under different current densities of I_K1_ and I_f_, with I_K1_ being reduced systematically by from between 60–100% (i.e., I_K1_ density at -80 mV changed from 0.396 to 0 pA/pF while the I_K1_ density in the original basal model is 0.99 pA/pF at -80 mV in I-V curve). The representative I-V relation curve under different inhibition of I_K1_ is shown in S1A Fig. I_f_ density was increased by from 0 to 10 folds with a basal value of -0.63 pA/pF at -80 mV in I-V curve (i.e., I_f_ density changed from 0 to -6.3 pA/pF at -80 mV). The representative I-V relation curve under different incorporation of I_f_ is shown in S1B Fig.

Two characteristics were used to quantify the state of membrane potentials generated by the ventricular pacemaker model: the continuity and validity of spontaneous APs. The continuity was used to quantify whether or not the automaticity of membrane potential could sustain with time; whilst the validity was used to characterize whether every automatic wave was biologically-valid or not. As such, we defined the following:

W: a valid wave. An action potential whose wave trough was less than -20 mV and wave crest was more than 20 mV could be considered as a valid wave.

αW, α<1: an incomplete wave.

R: a resting period lasting 1000 ms.

W^n^: the concatenation of n W’s.

(W R): the concatenation of W and R.

As such, the pacemaking behaviour of the I_K1_/I_f_ pacemaker model can be categorized as FIVE states whose definitions were as followings:

None pacemaking behaviour during the entire simulation period could be described as State-1:

$$R^{n},n\in N^{+}$$ (9)

Transient spontaneous pacemaking behaviour could be described as State-2:

$$(W^{m},R^{n}),m,n\in N^{+}$$ (10)

Bursting pacemaking behaviour could be described as State-3:

$${\left(W^{mi},R^{ni}\right)}^{M},i\in \left[1,M\right],M,m_{i},n_{i}\in N^{+}$$ (11)

Persistent pacemaking activity with periodically incomplete depolarization could be described as State-4:

$${\left(W^{m},\alpha W\right)}^{M},\alpha <1,m,M\in N^{+}$$ (12)

Stable pacemaking activity could be described as State-5:

$$W^{m},m\in N^{+}$$ (13)

With regard to the pacemaking ability, when the pacemaking behaviour was stable, the cycle length (CL) under varied I_K1_ and I_f_ was calculated. The CL was defined as the averaged wavelength of pacemaking activity over a period of simulation of more than 400 s, ensuring the accuracy of the computed CL. As the basal model was for VMs, a long simulation period was necessitated to achieve a completely stable pacemaking status and minimize the effect of the transition period.

### Characteristics of pacemaking during diastolic interval

The length of the diastolic interval (DI) is an important measure to characterize the pacemaking ability. In this study, we defined that DI as the time interval between the time of MDP (S2A Fig, t_1_) and the time when the membrane potential reaches at -55 mV (i.e., around the activation potential of I_CaL_) (S2A Fig, t_2_). The diastolic upstroke velocity during DI was defined as the change rate of the membrane potential, taking the following formulation:

$$\mathrm{diastolic}\;\mathrm{upstroke}\;\mathrm{velocity}=\frac{\mathrm{MDP}‐(‐55)}{t_{2}‐t_{1}}$$ (14)

The unit of diastolic upstroke velocity was V/s.

We evaluated the contributions of all of the inward currents to membrane potential during DI and found that the main inward currents which helped to depolarize membrane potential during DI are I_Na_, I_NaCa_ and I_f_. Their contribution can be described by an average integral during DI:

$$I_{\mathrm{in}}=\frac{\int_{t_{1}}^{t_{2}}\left(I_{\mathrm{Na}}+I_{\mathrm{NaCa}}+I_{f}\right)}{t_{2}‐t_{1}}$$ (15)

Similarly, the main outward currents which held membrane potential at diastolic potential during DI are I_K1_, I_NaK_, I_Kr_ and I_Ks_, the integral of which can be described as:

$$I_{\mathrm{out}}=\frac{\int_{t_{1}}^{t_{2}}\left(I_{K1}+I_{\mathrm{NaK}}+I_{\mathrm{Kr}}+I_{\mathrm{Ks}}\right)}{t_{2}‐t_{1}}$$ (16)

## Results

### Dynamic analysis in I_K1_/I_f_ parameter space

**Fig 1:**
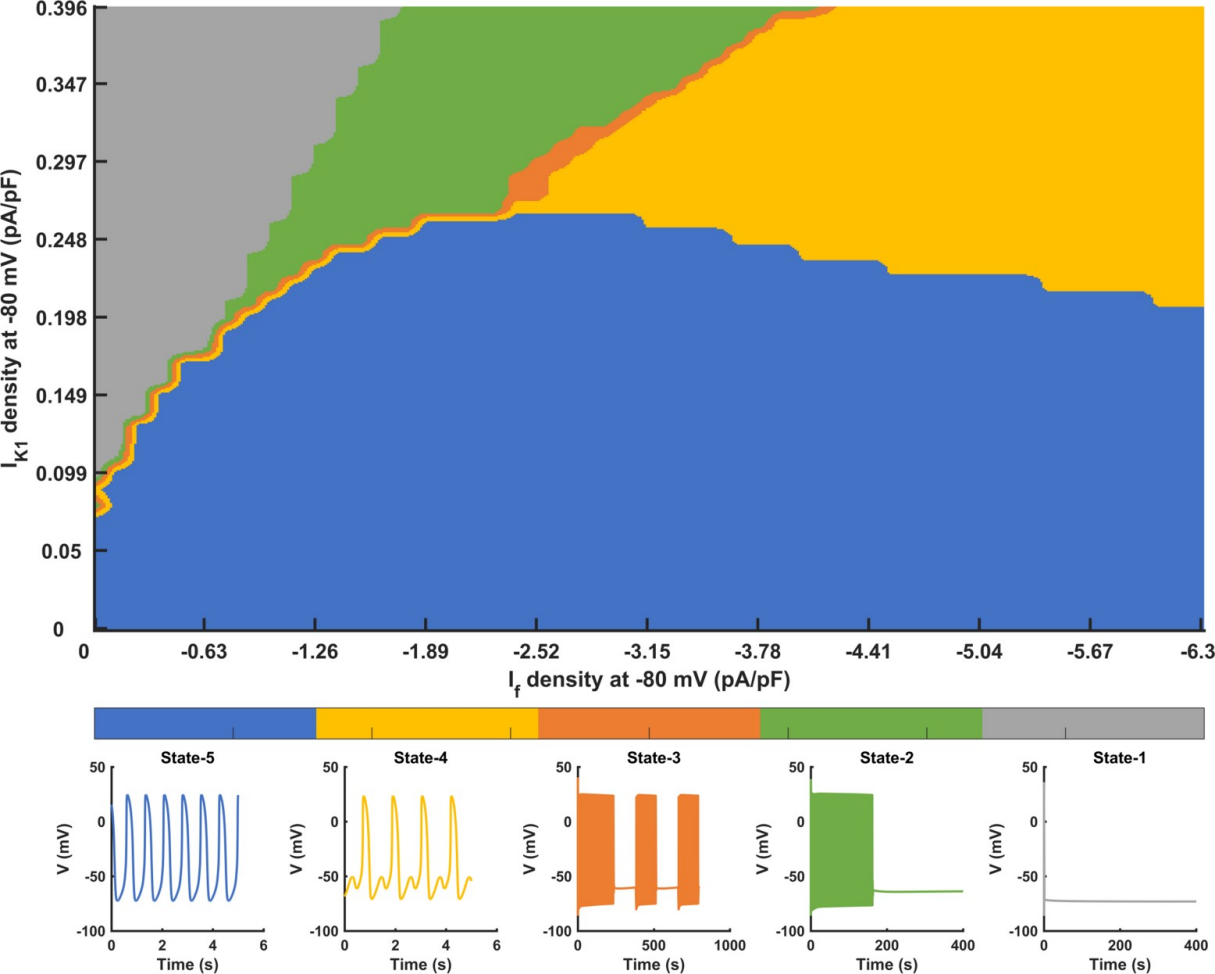
Dynamic behaviours of pacemaking action potentials in I_K1_/I_f_ parameter space. Blue: stable pacemaking activity (State-5); Yellow: persistent pacemaking activity with periodic incomplete depolarization (State-4). Orange: bursting pacemaking behaviour (State-3). Green: transient spontaneous pacemaking behaviour (State-2). Gray: no spontaneous pacemaking behaviour (State-1). In each category, the typical pacemaking action potentials are illustrated at the bottom panel.

Dynamic pacemaker AP behaviour was dependent on the balance of I_K1_ and I_f_ interactions. Simulations were conducted in the I_K1_/I_f_ parameter space to characterize this dependence. Results are shown in Fig 1. With differing combinations of I_K1_ and I_f_ density, five different regions for distinctive pacemaking dynamics could be discerned, including stable pacemaking activity (blue area, State-5 defined by Eq 13), intermittence of failed depolarization (yellow area, State-4 defined by Eq 12), bursting pacemaking behaviour (orange area, State-3 defined by Eq 11), transient pacemaking activity (green area, State-2 defined by Eq 10), and no automaticity (grey area, State-1 defined by Eq 9). In each category, representative membrane potentials are illustrated at the bottom panel of Fig 1.

With a fixed I_f_ density, alterations to I_K1_ could produce different types of pacemaking activities, and this also applied when I_K1_ was fixed whilst I_f_ was changed. When I_f_ density was fixed at a density between -0.63 and -2.52 pA/pF, with a 60–80% block of I_K1_ (i.e., I_K1_ density at -80 mV was in the range of 0.198–0.396 pA/pF), pacemaking activity was generated but with self-termination (Fig 1, green area). Then, a further reduction in I_K1_ or a slight increase in I_f_ induced bursting pacemaking behaviour, as shown by the orange area in Fig 1, which was between the boundaries marking the persistent automaticity and transient pacemaking activity regions. A further increase in I_f_ or suppression in I_K1_ could produce persistent automaticity (Fig 1, yellow and blue area). But when I_K1_ was greater than about 0.248 pA/pF, incomplete depolarization appeared periodically (Fig 1, yellow area). Finally, a stable and spontaneous pacemaking activity could be generated when I_K1_ was decreased to less than 0.248 pA/pF at -80 mV with I_f_ included (Fig 1, blue area).

To illustrate possible reasons responsible for the effect of each changed ion current density on modulating pacemaking states, we chose four typical cases to analyze the corresponding pacemaking mechanisms in different pacemaking states in the I_K1_/I_f_ parameter space. Cases are listed in Table 1.

**Table 1:** Typical cases and corresponding state.

CASE NO.	I_K1_ density (pA/pF)	I_f_ density (pA/pF)	State NO.
CASE 1	0.297pA/pF	-1.89 pA/pF	State-2
CASE 2	0.297 pA/pF	-2.52 pA/pF	State-3
CASE 3	0.297 pA/pF	-3.15 pA/pF	State-4
CASE 4	0.248 pA/pF	-3.15 pA/pF	State-5

### Initiation of transient spontaneous depolarization

**Fig 2:**
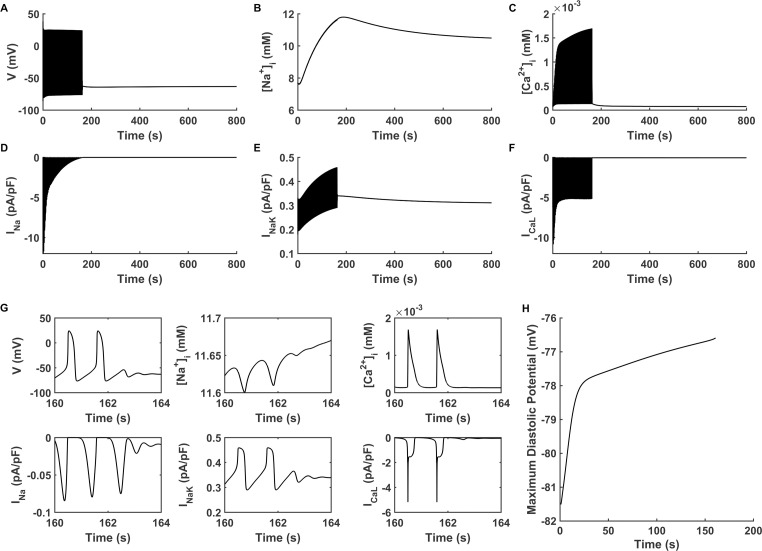
Transient spontaneous pacemaking behaviour. (A-F) Membrane potential (V), intracellular Na^+^ concentration ([Na^+^]_i_), intracellular Ca^2+^ concentration ([Ca^2+^]_i_), fast sodium current (I_Na_), Na^+^/K^+^ pumping current (I_NaK_) and L-type calcium channel current (I_CaL_) with the current densities of (I_K1_, I_f_) at (0.297 pA/pF, -1.89 pA/pF) (‘CASE 1’) during the entire simulating period of 800 s. (G) Expanded plots of (A-F) for the time course of pacemaking self-termination (160–164 s). H: Maximum diastolic potential of spontaneous pacemaking behaviour of (A).

In the basal VM cell model with the suppression of I_K1_ by 70% (the density of I_K1_ at -80 mV was 0.297 pA/pF), incorporation of I_f_ (with a current density of -0.63 pA/pF) was unable to depolarize the membrane potential and lead to spontaneous pacemaking activity because the excessive outward current of I_K1_ counteracted the inward depolarizing current. This state can be described by State-1 as shown in Eq 9. When the density of I_f_ was increased to -1.89 pA/pF, spontaneous depolarization was provoked at the beginning of the transition period, however, the automaticity self-terminated after 163 s (Fig 2A), showing a State-2 behaviour as described in Eq 10.

We analysed possible ion channel mechanisms responsible for unstable and self-terminating pacemaking APs with the current densities of (I_K1_, I_f_) at (0.297pA/pF, -1.89 pA/pF) (defined as ‘CASE 1’). Results in Fig 2 showed that during the time course of the spontaneous pacemaking, there were changes of intracellular ionic concentrations and the MDP. There was an accumulation of the intracellular Ca^2+^ concentration ([Ca^2+^]_i_, Fig 2C) during the time course of spontaneous pacemaking APs. Such an accumulation of [Ca^2+^]_i_ was because the automaticity in VMs shortened the DI between two successive APs, leaving insufficient time for Ca^2+^ in the cytoplasm to be extruded to restore to its initial value after each cycle of excitation. This consequentially led to overload in [Ca^2+^]_i_, which suppressed the extent of the activation degree of the L-type calcium current (I_CaL_, Fig 2F), especially during the phase 0 of the pacemaking action potential. Furthermore, the overloaded [Ca^2+^]_i_ increased the I_NaCa_ (S3A Fig) gradually with time, resulting in an elevated MDP (Fig 2H) that inhibited the activation degree of the fast sodium channel current (I_Na_, Fig 2D). Through the Na^+^ permeability of I_f_, extra Na^+^ flowed into the cytoplasm [53] during each of the APs. The increase of I_NaCa_ also accelerated the accumulation of the intracellular Na^+^ concentration ([Na^+^]_i_) to increase from 7.67 to 11.8 mM (Fig 2B). The increased [Na^+^]_i_ augmented the feedback mechanism of Na^+^/K^+^ pumping activity, by which the Na^+^/K^+^ pump current (I_NaK_) increased gradually with time (Fig 2E). All of these factors worked together, inhibiting the membrane potential to reach the take-off potential, leading to self-terminated automaticity at 163 s (Fig 2G).

It was also possible to generate automaticity in the model by fixing the current density of I_f_ at a low value, but with a further reduction in I_K1_ density. S4 Fig shows the results when I_f_ was held at -0.63 pA/pF and the current density of I_K1_ was reduced to 0.178 pA/pF. In this case, pacemaking activity appeared in the model, but the automaticity was unstable and self-terminated due to similar mechanisms as shown in Fig 2 for the increased-I_f_ situation.

### Bursting pacemaking behaviour

**Fig 3:**
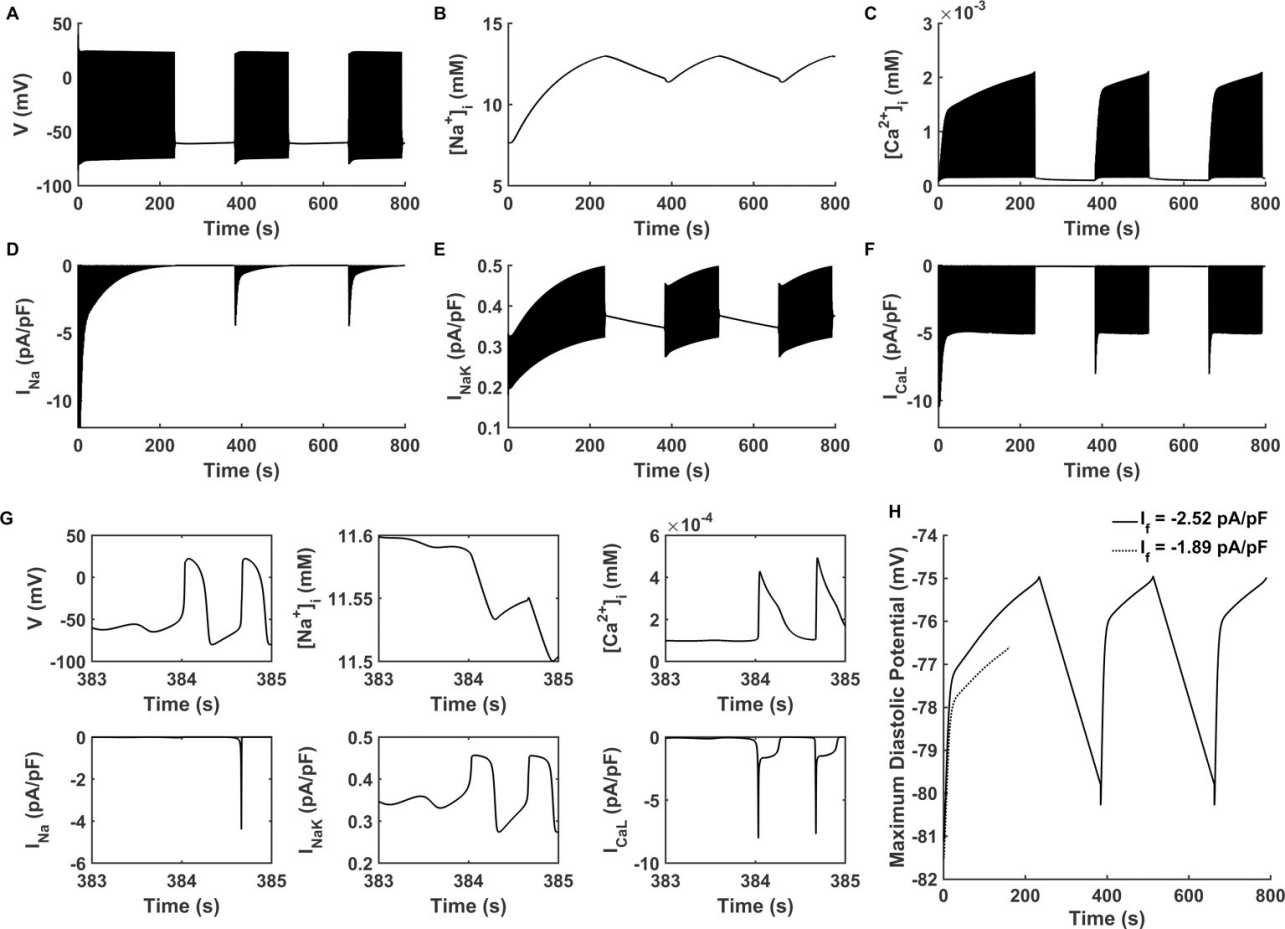
Bursting pacemaking behaviour. (A-F) Membrane potential (V), intracellular Na^+^ concentration ([Na^+^]_i_), intracellular Ca^2+^ concentration ([Ca^2+^]_i_), fast sodium channel current (I_Na_), Na^+^/K^+^ pumping current (I_NaK_) and L-type calcium channel current (I_CaL_) with the current densities of (I_K1_, I_f_) at (0.297 pA/pF, -2.52 pA/pF) (‘CASE 2’) during the entire simulating period of 800 s. (G) Expanded plots of (A-F) for the time course of pacemaking resumption (383–385 s). H: Maximum diastolic potential of automatic pacemaking activity when I_f_ is -2.52 and -1.89 pA/pF (‘CASE 2’ vs. ‘CASE 1’, solid and dotted line respectively).

Fig 3 shows the intermittent bursting behaviour, which is generated with a different combination of I_K1_ and I_f_ current densities in the model. In the figure, the current densities of (I_K1_, I_f_) were at (0.297 pA/pF, -2.52 pA/pF) (defined as ‘CASE 2’). Such kind of pacemaking state can be classified as State-3 (Eq 11).

In ‘CASE 2’, the spontaneous oscillation was unstable, characterized by self-termination and then resumption after a quiescent period (Fig 3A). During the first pacemaking stage (simulation period from 0 to 236 s in Fig 3), the self-termination was accompanied by the overload of [Na^+^]_i_, the accumulation of [Ca^2+^]_i_, which caused the reduction of I_Na_, the increase of I_NaK_ and the decrease of I_CaL_ (Fig 3B–3F). An increased [Na^+^]_i_, accumulated [Ca^2+^]_i_, reduced I_Na_, increased I_NaK_ and decreased I_CaL_ can also be observed in the pacemaking stage in Fig 2. This suggested that the underlying mechanisms responsible for the self-termination of the pacemaking APs were similar to those of ‘CASE 1’.

It is of interest to analyse the mechanism(s) for the resumption of the pacemaking APs after a long pause. It was shown that, during the time course of the quiescent interval (236–384 s in Fig 3A–3F), the intracellular Na^+^ (Fig 3B) continued to be extruded out of the cell by I_NaK_ (Fig 3E). As such, the [Na^+^]_i_ (Fig 3B) gradually decreased over time. The decrease in [Na^+^]_i_ led to a gradually reduced I_NaK_ over the time course of quiescence (Fig 3E), which decreased the suppressive effect of I_NaK_ on depolarization. *Via* I_NaCa_ (S3B Fig), the intracellular Ca^2+^ was kept to be extruded out of the cell, leading to a decreased [Ca^2+^]_i_ (Fig 3C). The reduction in [Ca^2+^]_i_ reduced the Ca-induced inactivation gate of I_CaL_, resulting in an increased I_CaL_. Ca^2+^ concentration in the sarcoplasmic reticulum ([Ca^2+^]_SR_) also decreased via a leakage Ca release (I_leak_) from SR to cytoplasm (S8C–S8D Fig) because of the reduced [Ca^2+^]_i_. Moreover, as compared with ‘CASE 1’, the increase in I_f_ helped to produce a more depolarized MDP (Fig 3H), allowing the membrane potential more easily to reach the take-off potential for initiation of the upstroke. During the quiescent stage, I_CaL_ and I_Na_ kept at a small magnitude (Fig 3D and 3F) because of the quiescent membrane potential at which little I_CaL_ and I_Na_ were activated. Until the Ca^2+^ oscillation produced a full course of action potential with a sufficiently depolarised MDP, I_f_ and I_Na_ was fully activated, facilitating the resumption of the spontaneous pacemaking activity at 385 s (Fig 3G). This process of self-termination and resumption repeated alternately, which constituted bursting behaviour.

### Persistent pacemaking activity

**Fig 4:**
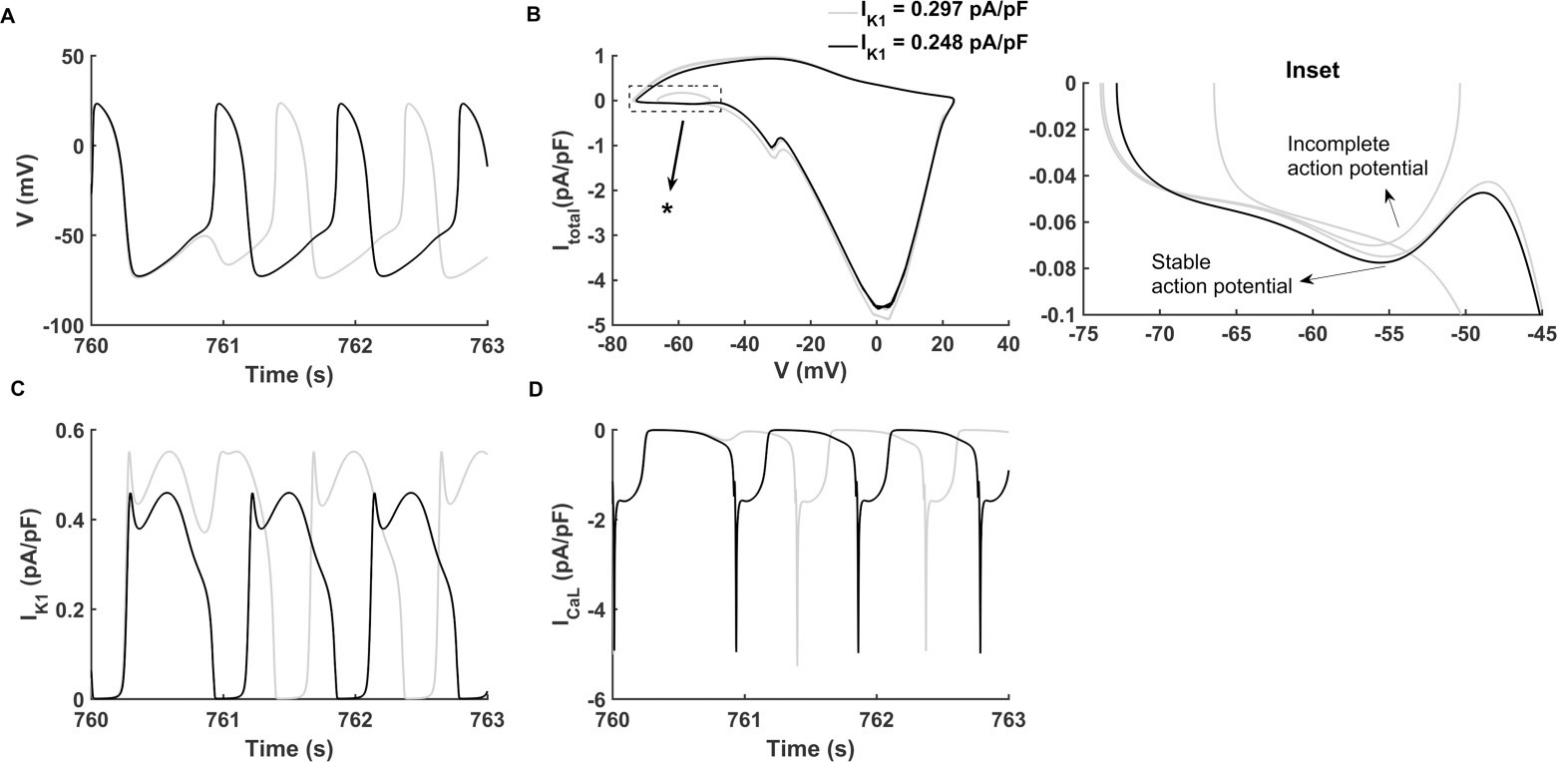
Persistent pacemaking activity. The current densities of (I_K1_, I_f_) of stable pacemaking activity and periodically incomplete pacemaking activity are at (0.248 pA/pF, -3.15 pA/pF) (‘CASE 4’, black lines) and (0.297 pA/pF, -3.15 pA/pF) (‘CASE 3’, grey lines) respectively. (A-D) The membrane potential (V), phase portraits of membrane potential against the total membrane channel current (I_total_), inward rectifier potassium channel current (I_K1_) and L-type calcium channel current (I_CaL_) during the simulating time course of 760–763 s. (Inset) Expanded plot during V from -75 to -45 mV and I_total_ from -0.1 to 0 pA/pF marked by the asterisk in (B).

A further increase in I_f_ ((I_K1_, I_f_) at (0.297 pA/pF, -3.15 pA/pF)) produced a series of persistent spontaneous APs. Results are shown in Fig 4 (grey lines) for APs (Fig 4A), together with a phase portrait of membrane potential (V) and total membrane channel current (I_total_) (Fig 4B), I_K1_ (Fig 4C), and I_CaL_ (Fig 4D). Although the spontaneous APs were sustained during the entire simulation period of 800 s, there were some incomplete depolarizations observed periodically (Fig 4A, grey line) which can be classified as State-4 according to Eq 12. This pacemaking situation was termed as ‘CASE 3’.

When the density of I_K1_ was further reduced to 0.248 pA/pF (Fig 4C, black line), a stable pacemaking activity was established (Fig 4A, black line), with an average CL of 895 ms and MDP of -72.63 mV. This kind of pacemaking state can be described as State-5 by Eq 13. In this condition, the pacemaking activity was robust and the pacing CL was close to that of the native human SAN cells (approximately 800–1000 ms [55]). We termed stable pacemaking activity with (I_K1_, I_f_) at (0.248 pA/pF, -3.15 pA/pF) as ‘CASE 4’.

In order to understand potential mechanism(s) underlying the genesis of incomplete pacemaking potentials in CASE 3, phase portraits of membrane potential against I_total_ for incomplete (grey line) and complete (black line) depolarization APs were plotted and superimposed for comparison, as shown in Fig 4B, with a highlight of phase portraits during the diastolic pacemaking potential range from -75 mV to about -45 mV being shown in the inset. In the case of incomplete depolarization (CASE 3), there was a greater I_K1_ (Fig 4C) that counteracted the inward depolarizing current, leading to a smaller I_total_ during the diastolic depolarization phase (see the grey line in Fig 4B and the inset). Consequentially the membrane potential failed to reach the take-off potential for the activation of I_CaL_ (Fig 4D, grey line), leading to an incomplete course of action potential (Fig 4A, grey line). In CASE 4, with a reduced I_K1_ (Fig 4C, black line), there was a greater I_total_ during the diastolic depolarization phase (Fig 4B and the inset, black line), which drove the membrane potential to reach the take-off potential for the activation of I_CaL_ (Fig 4D, black line), leading to the upstroke of the action potential.

The frequency for the appearance of the incomplete AP was dependent on the density of I_K1_. Incomplete depolarization occurred less frequently, with progressively smaller I_K1_. By way of illustration, the incomplete depolarization appeared once every three cycles with the I_K1_ density at 0.297 pA/pF in CASE 3 (S5A Fig), but this became once every five cycles when I_K1_ density was reduced to 0. 277 pA/pF I_K1_ (S5B Fig). This suggested that a large residual of I_K1_ in the VM model might result in the failure of complete depolarization.

### Pacemaking cycle length in I_K1_/I_f_ parameter space

**Fig 5:**
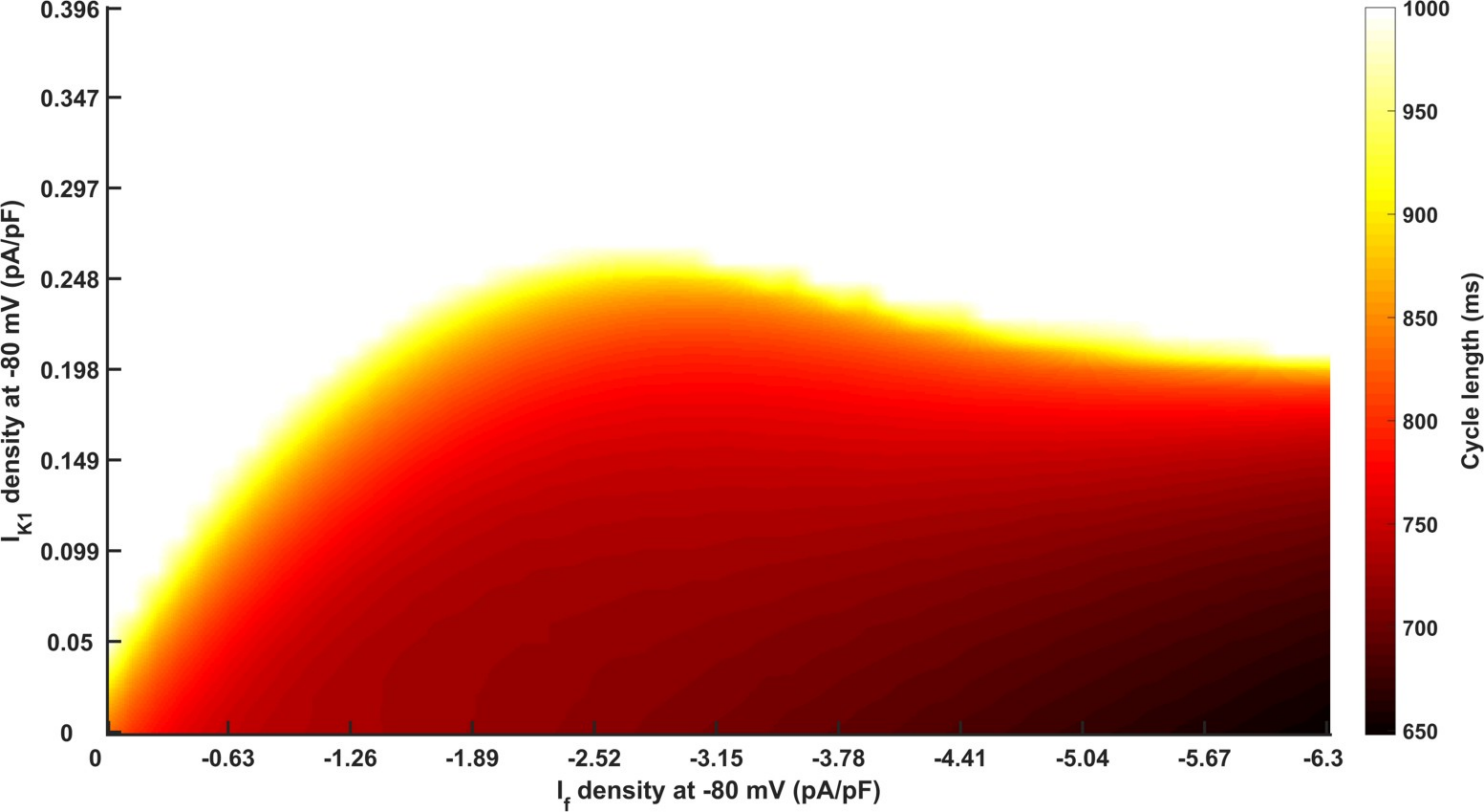
Measured cycle length in I_K1_/I_f_ parameter space. The density of I_K1_ is from 0 to 0.396 pA/pF and the density of I_f_ is from 0 to -6.3 pA/pF at -80 mV. The measured cycle length (CL) is coloured from 650 ms in dark red to 1000 ms in yellow. White means that there is no definitely measured pacemaking CL (i.e., the pacemaking action potential is not persistent or stable) or the pacemaking activity is ‘invalid’ (CL > 1000 ms).

A systematic analysis of the relationship between the calculated CL in the I_K1_ and I_f_ density parameter space is presented in Fig 5. In the figure, the measured CL was coloured from 650 ms in dark red to 1000 ms in yellow. In this study, we regarded persistent pacemaking action potential with CL 1000 ms or less as ‘valid pacemaking activity’ (corresponding to ‘State-5’ with CL < = 1000 ms in Fig 1), therefore, only the CLs of the valid pacemaking potentials are shown in Fig 5.

It was shown that a sufficient depression in I_K1_ (up to 75%; I_K1_ density < 0.248 pA/pF) was required to produce a stable pacemaking action potential with a ‘valid’ pacemaking frequency. With the increase of the I_K1_ inhibition level, the CL became shortened at all I_f_ densities considered. Also, the more that I_K1_ was inhibited, the less I_f_ was needed to provoke ‘valid’ spontaneous pacemaking activity. By contrast, the effect of I_f_ on the CL presented two phases, which was dependent on the I_K1_ density. When I_K1_ was less than 0.198 pA/pF, the pacemaking ability became robust with the increase in I_f_ density. However, when I_K1_ was increased from 0.198 to 0.248 pA/pF, an increase in I_f_ actually slowed the pacemaking activity, leading to an increased CL.

### Reciprocal role of I_f_ and I_K1_ in generating pacemaking APs

Further analysis was conducted to investigate the reciprocal role of reduced I_K1_ and increased I_f_ in generating pacemaking APs. This section analysed the role of I_f_ and I_K1_ in the parameter space which produced stable pacemaking activity (the ‘State-5’ in Fig 1) with the density of I_K1_ at 0.05 and 0.198 pA/pF. By sufficiently reducing I_K1_ to a density of 0.05 pA/pF alone (in the absence of I_f_), the model was able to generate stable spontaneous APs with a CL of 1011 ms (S6A and S6E Fig, dotted lines). In this case, the incorporation of I_f_ with a small density helped to boost the pacemaking activity and increase the pacemaking frequency. It was shown that with the incorporation of I_f_ at a density of -0.63 pA/pF, the CL was reduced by 233 ms, changing from 1011 ms to 778 ms (S6A Fig). Compared with the case of I_f_ absence, incorporation of I_f_—even with a small density—helped to depolarize cell membrane potential during the early DI phase (S6A and S6C Fig). Moreover, a similar phenomenon to Fig 2 was that the incorporation of I_f_ led to an accumulation of [Na^+^]_i_ (S6B Fig) and [Ca^2+^]_i_ (S6D Fig and Inset C). The accumulation of [Ca^2+^]_i_ increased I_NaCa_ (S6F Fig) which contributed to the depolarization of membrane potential. As a result, the incorporation of I_f_ facilitated genesis of spontaneous APs and shortened the DI significantly thus decreased the CL.

**Fig 6:**
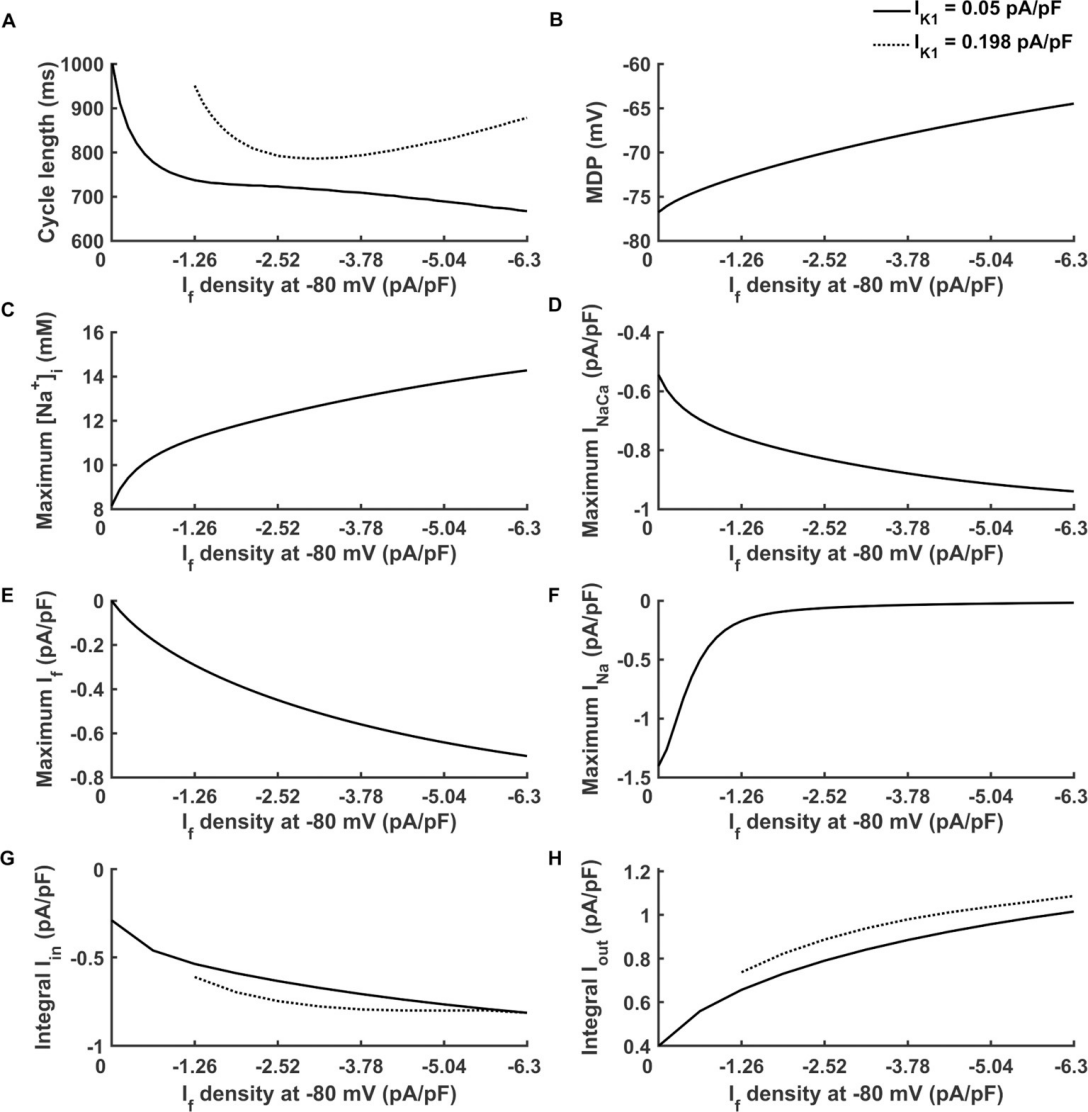
Effect of I_f_ density on the pacemaking cycle length under different I_K1_ density. (A) Change of cycle length with the increase of I_f_ from 0 to -6.3 pA/pF when I_K1_ density is 0.05 (solid line) and 0.198 pA/pF (dotted line). (B-F) Change of maximum diastolic potential (MDP), maximum intracellular Na^+^ concentration ([Na^+^]_i_), maximum Na^+^/Ca^2+^ exchange current (I_NaCa_), maximum funny current (I_f_) and maximum fast sodium current (I_Na_) during a pacemaking period with the increase of I_f_ from 0 to -6.3 pA/pF. (G-H) Change of the normalized total integral of main inward currents (Integral I_in_) and normalized total integral of main outward currents (Integral I_out_) during DI phase with the increase of I_f_ when I_K1_ density is 0.05 (solid line) and 0.198 pA/pF (dotted line). The inward currents include I_Na_, I_NaCa_ and I_f_; the outward currents include inward rectifier potassium channel current (I_K1_), Na^+^/K^+^ pumping current (I_NaK_), rapid delayed rectifier potassium channel current (I_Kr_) and slow delayed rectifier potassium channel current (I_Ks_).

With a fixed I_K1_ density of 0.05 pA/pF, the relationship between the computed CL of pacemaking APs and I_f_ density was shown in Fig 6A. In a range from 0 to -2.52 pA/pF, an increase in I_f_ density produced a marked decrease in the CL (Fig 6A), which was associated with an increase in the rate of membrane depolarization during the DI (diastolic depolarizing rate) (S2B Fig). In this range, an increase in I_f_ caused an accumulation of [Na^+^]_i_ (Fig 6C), which enhanced I_NaCa_ (Fig 6D). Particularly, there was a precipitous decline of I_Na_ when I_f_ was in the range from 0 to -1.26 pA/pF (Fig 6F). This was because the augmented I_f_ elevated the MDP of the action potential (Fig 6B), which affected the activation level of I_Na_. Furthermore, when I_f_ density was over -2.52 pA/pF, there was a less dramatic decrease in CL with an increase of I_f_ (Fig 6A). This was attributable to a reduced I_Na_ (Fig 6F) as a consequence of the gradual elevation of the MDP (Fig 6B). Another factor was that the maximum density of I_f_ was not increased linearly (Fig 6E) because of elevated MDP.

Depending on the I_K1_ density, the relationship between CL and I_f_ density could also be bi-phasic. With a small I_K1_ density (0.05 pA/pF), the measured CL decreased monotonically with the increase in I_f_ density (Fig 6A, solid line). However, with a large I_K1_ (0.198 pA/pF), the measured CL first decreased with an increased I_f_ density, but then increased with it when I_f_ density was greater than -3.15 pA/pF, implicating a slowdown in the pacemaking activity with the increase of I_f_ (Fig 6A, dotted line). The action potentials with the current densities of (I_K1_, I_f_) at (0.198 pA/pF, -3.78 pA/pF) and (0.198 pA/pF, -5.04 pA/pF) indicated that such a slowdown of pacemaking APs with an increased I_f_ was mainly due to the prolonged DI (S1 Text). The extra I_f_ accelerated pacemaking activity, leaving insufficient time for intracellular Ca^2+^ to be extruded, which resulted in a higher amplitude of [Ca^2+^]_i_ (Fig A in S1 Text). As a consequence, I_NaCa_ amplitude increased, leading to an increased [Na^+^]_i_, and finally an increased outward I_NaK_ (Fig B in S1 Text). At the same time, I_f_ elevated the MDP (Table A in S1 Text), so the amplitude of I_Na_ decreased (Fig B in S1 Text). The changes in these currents caused a prolonged CL at a great I_f_ density. This indicated that the bi-phasic effect of I_f_ may be attributable to the delicate balance between inward currents and outward currents during the DI phase. To extend this hypothesis to the whole I_f_ parameter space, I_in_ and I_out_ were defined as the total integral of three dominant inward currents during the DI phase (I_Na_, I_NaCa_ and I_f_) (Eq 15) and the total integral of four dominant outward currents during the DI phase (I_K1_, I_NaK_, I_Kr_ and I_Ks_) (Eq 16). At a low I_K1_ density of 0.05 pA/pF, with the increase in I_f_ density, there was a monotonic decrease of CL (Fig 6A, solid line). However, at a large I_K1_ density (e.g., 0.198 pA/pF), more than -3.15 pA/pF I_f_ resulted in an increased CL (Fig 6A, dotted line). This observation can be explained by the unsaturated I_in_. The density of I_in_ was an integrated consequence of MDP, the activation degree of I_Na_ and I_f_, as well as the density of I_NaCa_. At 0.05 pA/pF I_K1_ density, I_out_ was small, therefore I_Na_, I_NaCa_ and I_f_ was saturated (i.e., I_in_ was saturated). However, when I_K1_ was large, increased I_out_ led to a more negative MDP which resulted in an increased I_in_ due to more activated I_Na_, I_NaCa_ and I_f_. But I_in_ reached at a asymptotic value after I_f_ density was greater than -3.15 pA/pF (Fig 6G, dotted line) because the a similar level of the diastolic [Ca^2+^]_i_ (i.e., minimum [Ca^2+^]_i_) at different I_K1_ caused a decreased normalized integral of I_NaCa_ (Fig C in S1 Text), therefore I_in_ was not saturated at large I_K1_ (Fig 6G, dotted line). Consequentially, the I_out_ outbalanced the I_in_, leading to a prolonged DI that increased the CL at large I_K1_.

### Incorporation of I_CaT_ in I_K1_/I_f_ pacemaker model

The incorporation of I_CaT_ in the I_K1_/I_f_ pacemaker model with the current densities of (I_K1_, I_f_) at (0.297 pA/pF, -0.63 pA/pF) induced automaticity in non-pacemaking cell model (S7A Fig). It was shown that I_CaT_ initiated the oscillation of Ca^2+^ in the cytoplasm and the SR (S7B and S7C Fig) in the non-pacemaking cell model, activating the I_NaCa_ and I_CaL_ (S7D and S7E Fig).

However, when I_CaT_ was incorporated into a stable I_K1_/I_f_ pacemaker model with the current densities of (I_K1_, I_f_) at (0.099 pA/pF, -0.63 pA/pF), an increased pacemaking CL was observed (853 ms vs. 950 ms, S8A Fig). The reason was that the addition of I_CaT_ in the model affected the Ca^2+^ dynamics, resulting in an accumulation of [Ca^2+^]_i_ as well as [Ca^2+^]_SR_, leading to an increased I_NaCa_ and greater inactivation of I_CaL_ (S8C–S8F Fig). An increased I_NaCa_ consequently caused an accumulation of [Na^+^]_i_, leading to an increased outward I_NaK_ (S8G Fig). More importantly, an increased I_NaCa_ elevated the MDP, leading to a less activated I_Na_ and I_f_ (S8H and S8I Fig). These together with an increased I_NaK_ produced a prolonged CL.

## Discussion

### Summary of major findings

Biological experiments provided possibilities to transform non-pacemaking CMs or stem cells into bio-pacemaker cells by regulating the expression of I_f_ [13,28,29,36,37] or I_K1_ [11,20,21]. Computationally, bifurcation theory was used to analyse the effect of the density of an individual ion channel current on the membrane potential of a cardiac cell [47,50], by which decisive currents in pacemaking initiation can be screened out. The interaction between multiple ion channels in cardiac pacemaking has also been considered in some prior studies. Lakatta et al. [51] investigated the effect of multiple component ion channels on cardiac pacemaking by identifying numerically minimal ensembles of ion channels in the SAN model. Their study provided a simplification of the model which may be suitable for further bifurcation analysis. However, due to the simplification, the model does not reflect the whole ionic dynamics of cardiac cells. In another study, Kurata et al. [50] simulated the combined action of I_f_ and I_CaT_, I_st_ or I_CaL_ with three specific values of I_CaT_, I_st_ or I_CaL_ considered. That study also suggested that there was a threshold of I_K1_ below which automaticity can be induced in I_f_-incorporated ventricular model, but potential underlying ionic mechanisms on how balanced I_K1_/I_f_ modulates the stability of the pacemaking activity was not shown. Considering the biological possibility that the superiority of combined action of I_K1_ and I_f_ [10,32,44] and unclear interaction between I_K1_ and I_f_ in the initiation of stable pacemaking activity in a whole cardiac cell model, the present study developed a virtual bio-engineered pacemaking cell model based on a human ventricular model by downregulating of I_K1_ and incorporating I_f_. In comparison with previous computational modelling studies on bio-pacemakers [50,51], the present study provides new insights into understandings of 1) the dynamic regulation of ionic concentrations and ionic channel currents in I_K1_/I_f_-induced ventricular pacemaker; 2) the interaction between I_K1_ and I_f_ in the genesis of pacemaking action potentials; and 3) the stability of pacemaking potentials in wide I_K1_/I_f_ parameter space.

The present I_K1_/I_f_ pacemaker model can initiate robust and stable pacemaking activity by balancing the actions of reduced I_K1_ and increased I_f_, though the effect of each manipulation on pacemaking activity is different. While the action of a reduced I_K1_ on the pacemaking activity is mono-phasic, that of an increased I_f_ is biphasic. It was shown that inhibiting I_K1_ promotes pacemaking ability and stability. The incorporation of I_f_ at an appropriate level promotes pacemaking activity, but an excessive I_f_ might result in abnormal pacemaking activity accompanied by abnormal intracellular ionic concentrations (such as pacemaking activity with periodically incomplete depolarization) or weak pacemaking ability, which could be proarrhythmic. As a result, the reciprocal interaction between I_K1_ and I_f_ is crucial for producing stable spontaneous pacemaking activity in VMs. In the present model, specific I_f_ density at different I_K1_ densities or vice versa was suggested.

This study demonstrated the feasibility of creating VM-based pacemaker cells by downregulating I_K1_ and upregulating I_f_ and provided evidence of the superiority of I_K1_/I_f_-based pacemaker model theoretically. The results of this study may be useful for optimizing the future design of engineered bio-pacemakers.

### Role of I_K1_ suppression on pacemaking activity

Simulation results that suppressing I_K1_ by 75% - 100% can initiate stable pacemaking behaviour are in consistence with those of experimental findings, where it has been found that more than 80% inhibition of I_K1_ was required to produce a pacemaking phenomenon in guinea-pig’s ventricular cavity [11,21]. It is also in agreement with previous bifurcation analyses in showing that it required I_K1_ to be reduced to at least 15% of the control value to transform a VM cell model to be auto-rhythmic [47]. And a complete block of I_K1_ produced a spontaneous pacemaking activity with a CL of 795 ms [47], close to 833 ms when I_K1_ was completely suppressed in the present study. The effect of I_K1_ suppression on pacemaking activity was monotonic in our pacemaker model. The more the I_K1_ is blocked, the faster the pacemaking activity is with all I_f_ densities considered. Similar results have also been observed in another human ventricular cell model [56] based on modifications of the O’Hara and Rudy model (ORD model) [57] (Fig A in S2 Text, solid and dotted lines).

Though our simulation results suggest an important role of sufficient suppression of I_K1_ in generating persistent and stable pacemaking APs, it is noteworthy that the deficiency of I_K1_ has been reported to be lethal for adult rodents [58]; and loss function of *Kir2* gene may prolong QT intervals as well as cause Andersen’s syndrome [59]. Consequently, suppression of I_K1_ from VM for generating a biological pacemaker may only be suitable when applied to highly localized, designated ‘pacemaker’ regions.

### Role of I_f_ on pacemaking activity

I_f_ has been shown to play an important role in generating pacemaking APs in both native [13,22,28,29,36,37] and engineered pacemakers [43]. Experimentally it has been shown that high expression of *HCN2* can initiate spontaneous beats in neonatal rat VMs [22,36] and improve spontaneous beats in rabbit CMs [13]. *HCN4* incorporation by the expression of *TBX18* can also initiate spontaneous pacemaking activity in both rodent VMs [10] and porcine VMs [12].

In the present study, I_f_ helped to promote pacemaking activity, *via* its action of depolarization during the diastolic depolarization phase as well as its action on the intracellular ion concentrations. The inclusion of I_f_ in the VM cell model caused the accumulation of [Na^+^]_i_ by Na^+^ channel of I_f_ [53]. The enhanced pacemaking activity caused by extra I_f_ also induced the accumulation [Ca^2+^]_i_ because there was not enough time to extrude Ca^2+^ from the cytoplasm [60]. The accumulated [Ca^2+^]_i_ increased I_NaCa_, which promoted membrane potential depolarization especially during the early stage of DI (S6 Fig). Such a promoting action of I_f_ in bio-pacemaking can also be seen in another independent model as shown in S2 Text.

The increase in I_f_ density can enhance the automaticity in most cases. However, the effect of I_f_ on the pacemaking activity was observed to be bi-phasic. When it was increased to be over a threshold, excessive I_f_ resulted in an elevated MDP (Fig 6), which caused a reduced activation of I_f_ and I_Na_, leading to a slowdown of the ability of pacemaking activity. This phenomenon occurred when the I_K1_ was not suppressed sufficiently. The impairing effect of excessive I_f_ on pacemaking APs was also observed in another ventricular pacemaker model based on modification of the ORD model [56] (S2 Text). It was shown that a greater increase in I_f_ density even terminated pacemaking activity (Fig B in S2 Text). The possible impairing effect of I_f_ on I_Na_ was verified by the fact that in the bio-pacemaker induced by *HCN2* expression [61], co-expression of the skeletal muscle sodium channel 1 (*SkM1*), in order to hyperpolarize the action potential threshold, helped to counterbalance the negative effect of I_f_ overexpression, producing an accelerated depolarization phase. In fact, in the original TP06 model, the peak amplitude of I_Na_ was about -300 pA/pF at the resting potential of -86.2 mV [54]. In our pacemaker model, the peak amplitude of I_Na_ was significantly reduced because of the elevated MDP. This suggested that to counterbalance the elevated MPD and the reduced I_Na_, an increase in the channel expression of I_Na_ may help to produce an enhanced pacemaker. This might be simulated by increasing I_Na_ conductance in the model study. Furthermore, when I_K1_ was large, an increase in I_f_ even lengthened pacemaking period or caused unstable pacemaking behaviour (Fig 1). This simulation result is in agreement with a previous biological experimental study that observed arrhythmicity when acute *HCN* gene was expressed [42]. Another experimental study showed that *HCN2*-expression caused an excessive increase in the basal beating rate [62]. In our model, it has been also observed that excessive I_f_ may cause an overly fast pacemaking rate.

### Reciprocal interaction between I_K1_ and I_f_

Our study demonstrates that the reciprocal interaction between I_K1_ and I_f_ plays a crucial role in creating stable and persistent pacemaking. Only an optimal combination of I_K1_ and I_f_ can initiate stable pacemaking activity. In the present pacemaker model, the greater the degree of I_K1_ suppression, the smaller was the I_f_ density required for the generation of spontaneous oscillation (Fig 1). And modulation of the two currents simultaneously helps to create a physiologically-like pacemaker that is better than that produced by manipulating I_K1_ or I_f_ alone (Fig 5). Such observation of reciprocal interaction between I_K1_ and I_f_ in pacemaking is consistent with previous experimental observations. Previous studies have shown that although suppressing I_K1_ [11,21], or incorporating sufficient I_f_ [22] alone was able to initiate pacemaking activity in VM cells, a pacemaker constructed by *TBX18* showed greater stability, due to its combined actions of I_K1_ reduction and I_f_ increase [10]. Another experiment in porcine VMs [12] also indicated that *TBX18* expression did not increase the risk of arrhythmia, which means that a mixed-current approach is probably a superior means of producing a bio-pacemaker. Experiments in a *Kir2*.*1*/*HCN2* HEK293 cell [45] and *Kir2*.*1*/*HCN4* [43] showed that I_K1_ may actually recruit more I_f_ by activating current at more negative membrane voltages because I_K1_ was the only hyperpolarizing current in these experiments. Our simulations, however, did not yield such a result because the interaction of other outward currents (such as I_NaK_, I_Kr_ and I_Ks_) contributed to the hyperpolarization of membrane potential and helped the activation of I_f_. We thought an integrated action between all of ionic currents in cardiac cells should be considered, rather than evaluated specific ionic currents in a partial model.

In addition, simulation results indicated that I_K1_ expression level may influence the I_f_’s effect on the pacemaking activity (Fig 6A). Excessive I_K1_ hindered I_f_’s ability to modulate pacemaking activity. This further showed that the balanced expression of I_K1_ and I_f_ affected the balance between the inward and outward currents during the diastolic depolarization phase, thus affected the membrane potential state and the pacemaking CL of the pacemaker. An experiment showed a coincident result that the expression of *HCN2* in adult rat VMs could not cause spontaneous beats due to the high expression of I_K1_ [22], but in neonatal rats, the I_K1_ was less so that expressing *HCN2* could provoke automaticity. Similarly, such a dynamic balance between the inward and outward currents during the repolarization and the diastolic depolarization phase was also affected by other repolarization currents, such as I_Kr_, I_Ks_ and I_NaK_ etc. Possible effects of modulating these repolarization currents on the bio-pacemaking warrants further studies in the future.

The present I_K1_/I_f_-induced pacemaker model exhibited greater robustness than I_K1_-based or I_f_-based pacemaker models. In the I_K1_-based model, the range of I_K1_ density that could initiate spontaneous beatings was from 0 to 0.0246 pA/pF, while in I_K1_/I_f_-based pacemaker model, this value extended to 0.248 pA/pF (Fig 1). The superiority of I_K1_/I_f_-based pacemaker model than I_f_-based pacemaker model seemed to be more distinct. Incorporating I_f_ alone at a high density of -6.3 pA/pF could not provoke any spontaneous beating, but combining with the suppression of I_K1_, small incorporation of I_f_ helped to ignite automaticity (Fig 1). The flexibility of this system also reflected in the easy modulation of CL *via* manipulating I_K1_ and I_f_ density (Fig 5).

Compared with the human SAN cell model developed by Fabbri et al. [53] and human SAN cell [64], the action potential generated by the I_K1_/I_f_-induced pacemaker model had a longer action potential duration at 90% (APD90) and a more negative MDP when the CL was similar (see Table 2). Such differences may be attributable to the fact that there are regional differences in the functional expression of ionic currents between the SAN and ventricular myocytes [63]. In the presented I_K1_/I_f_-induced pacemaker model, though we have reduced I_K1_ and incorporated I_f_ to a similar level of ion channel current densities as the SAN, other ionic currents in the present I_K1_/I_f_-induced pacemaker model inherited the same channel properties of VMs cell model, causing different pacemaker behaviour in the I_K1_/I_f_ biological pacemaker model as compared to the SAN model.

**Table 2:** Comparison of pacemaker behaviours between the SAN cell and the bio-pacemaker cell.

Type	CL (ms)	APD90 (ms)	MDP (mV)
Human SAN Experiment [64]	828 ± 21	143.5 ± 49.3	-61.7 ± 6.1
Human SAN Model [53]	814	161.5	-58.9
Human Bio-pacemaker Model*	817	299	-75.1683

* The current densities of (I_K1_, I_f_) is (0.1 pA/pF, -0.882 pA/pF).

### Ca^2+^ dynamics in I_K1_/I_f_ pacemaker model

There is still debate about the relative role of two pacemaking mechanisms of membrane clock (I_f_) and Ca^2+^ clock [65]. A biological experiment demonstrated that Ca^2+^-stimulated adenylyl cyclase AC1 can promote pacemaking ability in *HCN2*-expressed left bundle branches [62]. A model study [66] that evaluated the synergism between Ca^2+^ clock and membrane clock in SAN central cell, also showed that the synergistic system was more robust and flexible. Another study [67] showed that VMs may also have Ca^2+^ clock, which provided a probability for the creation of Ca^2+^ clock-based bio-pacemaker. The role of Ca^2+^ dynamics in bio-pacemaker was also shown in our I_K1_/I_f_ pacemaker model. As shown in Fig 3G, the resumption of pacemaking activity in bursting behaviour was provoked by the oscillation of [Ca^2+^]_i_. This indicated that the Ca^2+^ dynamics played an important role in the creation of bio-pacemaker, which warrants further study.

Furthermore, considering the role of I_CaT_ in the genesis of pacemaking APs in native SAN cells, a theoretical investigation of potential role(s) of I_CaT_ in the bio-pacemaker was conducted using the I_K1_/I_f_-modulated pacemaker model. It was shown the effect of I_CaT_ had dual aspects. On one hand, the incorporation of I_CaT_ might promote the pacemaking ability of ventricular pacemaker [50]. by initiating Ca^2+^ oscillation thus producing spontaneous beatings in quiet pacemaker model. On the other hand, the incorporation of I_CaT_ might affect the MDP, leading to secondary actions on the homeostasis of ion concentrations, as well as ion channel currents including I_Na_, I_f_, I_NaCa_ and I_NaK_, which slowed down the pacemaking activity.

### Limitations

Limitations of the human VMs model we used in this study has been described elsewhere [54]. In this study, the I_f_ formulation of human SAN [53] was incorporated into the original VMs model. The properties of I_f_, including the conductance of I_f_, the half-maximal activation voltage (V_1/2_) and time constants of the activation, may present species-dependence. In the present version, we only consider the conductance of I_f_ but have not discussed other properties of I_f_. Moreover, in this study, we only investigated the pacemaking action potential at the single-cell level, without considering the intercellular electrical coupling between pacemaker cells as presented in the SAN tissue. These limitations are now being addressed for future versions of the model. In addition, bio-pacemaker models developed from other cardiac cell types, such as atrial myocytes, warrant future studies. Additionally, the other pacemaking-related currents in native SAN cells, such as I_Na_ and I_st_, could also be adjusted for creating stable bio-pacemaker.

One of possible advantages of bio-pacemaker over the traditional electronic pacemaker is at its possible sensitivity to autonomic regulation. It is of interest to study how the pacemaking action potentials are modulated by autonomic regulation by β-Adrenergic receptor stimulation or cholinergic receptor stimulation [10], which warrants further future investigation.

It is necessary to highlight these limitations, they nevertheless do not affect our conclusions on the underlying pacemaking mechanisms of engineered bio-pacemaker cells, especially regarding the reciprocal interaction of I_K1_ and I_f_ for a robust bio-pacemaker in modified VMs.

## Supporting information

S1 Fig I-V relation of I_K1_ and I_f_ with different expression level. S_K1_ and S_f_ are defined as scaling factors used to simulate the change of I_K1_ and I_f_ expression level. (A) The I-V curve of I_K1_ with S_K1_ of 1, 0.4, 0.1 that gives I_K1_ densities in the I-V curve at -80 mV 0.99, 0.396 and 0.099 pA/pF respectively. (B) The I-V curve of I_f_ with S_f_ of 1, 5, 10 that gives I_f_ densities in the I-V curve at -80 mV -0.63, -3.15 and -6.3 pA/pF respectively.
(TIF)

S2 Fig Change of diastolic depolarizing rate with the increase of I_f_ density. (A) Definition of diastolic depolarizing rate. MDP: maximum diastolic potential; t_1_: the time when membrane potential is MDP; t_2_: the time when potential arrives -55 mV (i.e., around the activation potential of the I_CaL_). (B) Change of diastolic depolarizing rate with the increase of I_f_ density from 0 to -6.3 pA/pF when I_K1_ density at -80 mV is at 0.05 pA/pF.
(TIF)

S3 Fig Ca dynamic of the transient and bursting pacemaking behaviour. (A) Na^+^/Ca^2+^ exchange current (I_NaCa_) during the entire simulating period of 800 s with the current densities of (I_K1_, I_f_) at (0.297pA/pF, -1.89 pA/pF). (B-D) Na^+^/Ca^2+^ exchange current (I_NaCa_), Ca^2+^ concentration in sarcoplasmic reticulum ([Ca^2+^]_SR_) and leakage current from SR to cytoplasm (I_leak_) during the entire simulating period of 800 s with the current densities of (I_K1_, I_f_) at (0.297 pA/pF, -2.52 pA/pF).
(TIF)

S4 Fig Transient spontaneous pacemaking behaviour. Membrane potential (V) during the entire simulation period of 400 s with the current densities of (I_K1_, I_f_) at (0.178 pA/pF, -0.63 pA/pF).
(TIF)

S5 Fig Persistent pacemaking activity with periodically incomplete depolarization at different densities of I_K1_. (A-B) Membrane potential (V) with the current densities of (I_K1_, I_f_) at (0.297 pA/pF, -3.15 pA/pF) and (0.277 pA/pF, -3.15 pA/pF) during simulating time course of 360–370 s.
(TIF)

S6 Fig Positive effect of I_f_ on pacemaking ability. (A-F) The membrane potential (V), intracellular Na^+^ concentration ([Na^+^]_i_), “funny” current (I_f_), intracellular Ca^2+^ concentration ([Ca^2+^]_i_), inward rectifier potassium channel current (I_K1_) and Na^+^/Ca^2+^ exchange current (I_NaCa_) during simulating time course of 400–403 s when the current densities of (I_K1_, I_f_) are at (0.05 pA/pF, 0 pA/pF) and (0.05 pA/pF, -0.63 pA/pF) (dotted and solid line respectively). (Inset A-B) Expanded plots of [Na^+^]_i_ traces for the time course marked by the horizontal brackets with asterisks in (B). (Inset C) The change of [Ca^2+^]_i_ with simulating time course of 0–100 s.
(TIF)

S7 Fig Positive effect of incorporating I_CaL_ on quiet I_K1_/I_f_ pacemaker model. (A-F) Membrane potential (V), intracellular Ca^2+^ concentration ([Ca^2+^]_i_), Ca^2+^ concentration in sarcoplasmic reticulum ([Ca^2+^]_SR_), Na^+^/Ca^2+^ exchange current (I_NaCa_), L-type calcium channel current (I_CaL_) and T-type calcium channel current (I_CaT_) with the current densities of (I_K1_, I_f_) at (0.297 pA/pF, -0.63 pA/pF) during the simulating period of 0–20 s.
(TIF)

S8 Fig Side effect of incorporating I_CaL_ on stable I_K1_/I_f_ pacemaker model. (A) Membrane potential (V) during the simulating period of 300–302 s. (B-H) Membrane potential (V), intracellular Ca^2+^ concentration ([Ca^2+^]_i_), Ca^2+^ concentration in sarcoplasmic reticulum ([Ca^2+^]_SR_), Na^+^/Ca^2+^ exchange current (I_NaCa_), L-type calcium channel current (I_CaL_), Na^+^/K^+^ pumping current (I_NaK_), fast sodium current (I_Na_) and “funny” current (I_f_) with the current densities of (I_K1_, I_f_) at (0.099 pA/pF, -0.63 pA/pF) during the simulating period of 0–20 s.
(TIF)

S1 Text Prolonged cycle length at greater I_f_ density. (DOC)

S2 Text Model-dependence test. (DOC)

S1 Code Biological pacemaker cell model. (CPP)
