# Supplementary figures and images for: Reciprocal interaction between IK1 and If in biological pacemakers: A simulation study
Source: PLoS Comput Biol. 2021 Mar 10;17(3):e1008177. doi: 10.1371/journal.pcbi.1008177 (PMC7984617; doi:10.1371/journal.pcbi.1008177)

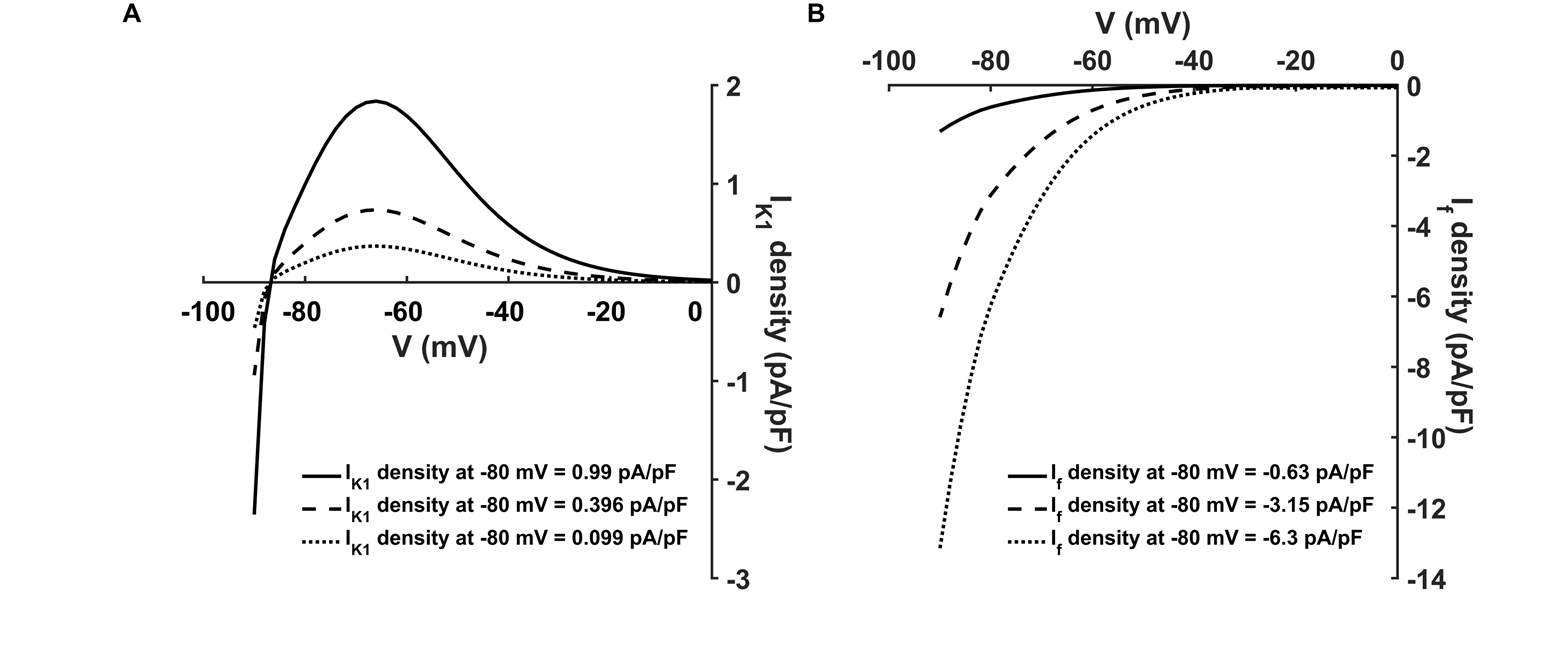

Supplement: S1 Fig — SK1 and Sf are defined as scaling factors used to simulate the change of IK1 and If expression level. (A) The I-V curve of IK1 with SK1 of 1, 0.4, 0.1 that gives IK1 densities in the I-V curve at -80 mV 0.99, 0.396 and 0.099 pA/pF respectively. (B) The I-V curve of If with Sf of 1, 5, 10 that gives If densities in the I-V curve at -80 mV -0.63, -3.15 and -6.3 pA/pF respectively. (TIF) [file pcbi.1008177.s001.tif]

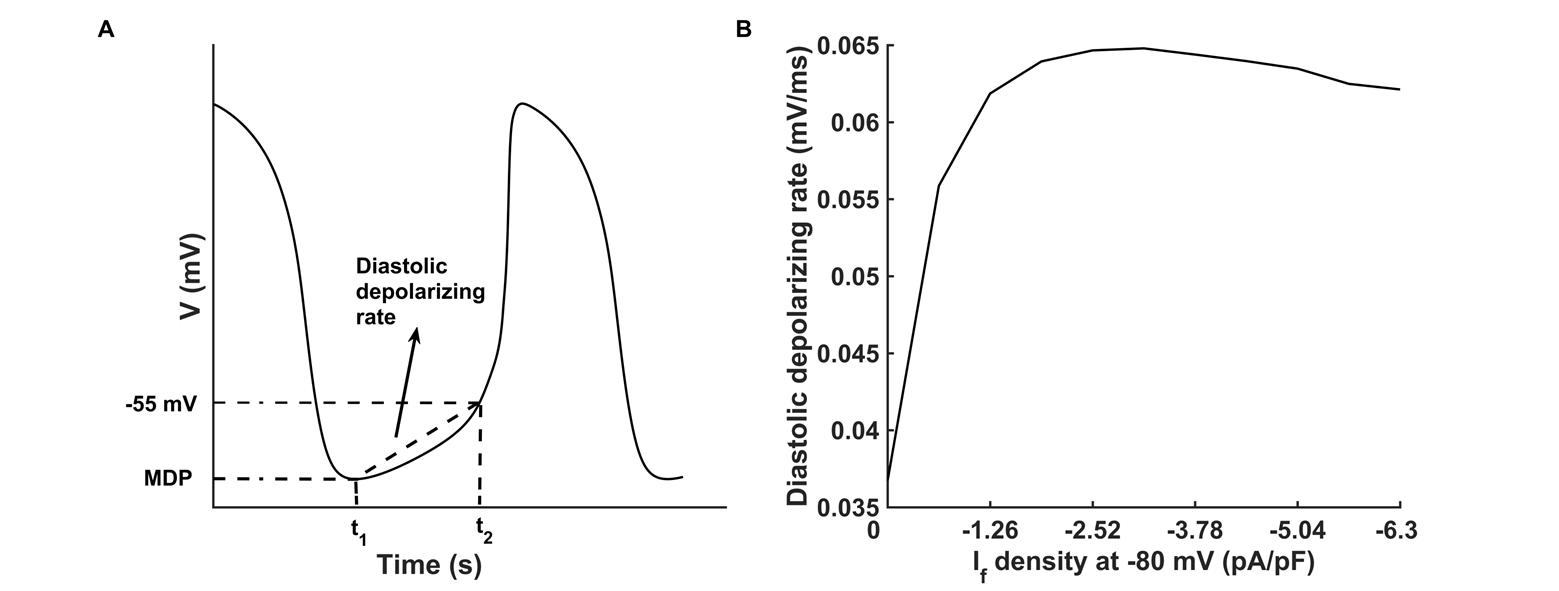

Supplement: S2 Fig — (A) Definition of diastolic depolarizing rate. MDP: maximum diastolic potential; t1: the time when membrane potential is MDP; t2: the time when potential arrives -55 mV (i.e., around the activation potential of the ICaL). (B) Change of diastolic depolarizing rate with the increase of If density from 0 to -6.3 pA/pF when IK1 density at -80 mV is at 0.05 pA/pF. (TIF) [file pcbi.1008177.s002.tif]

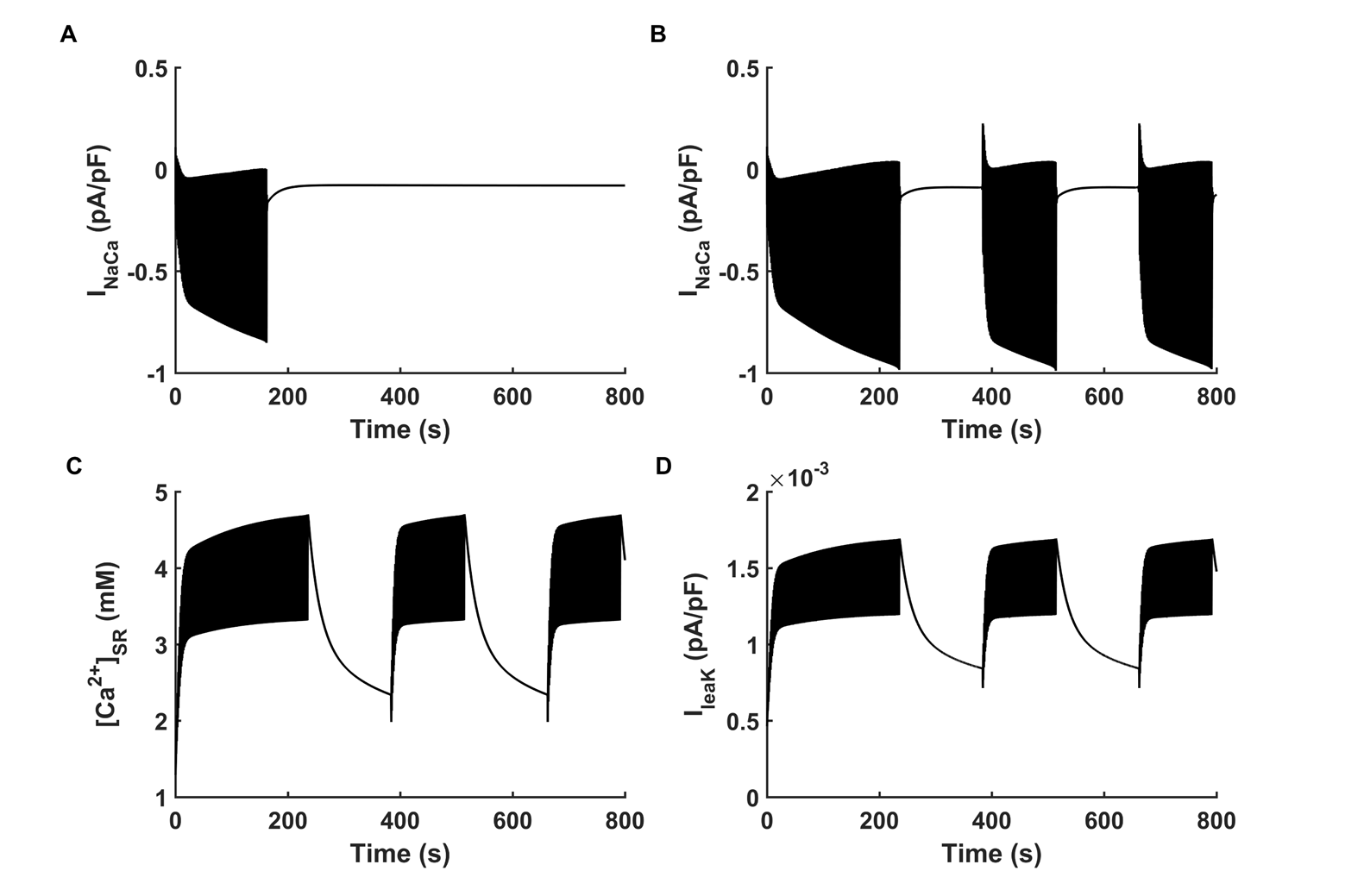

Supplement: S3 Fig — (A) Na+/Ca2+ exchange current (INaCa) during the entire simulating period of 800 s with the current densities of (IK1, If) at (0.297pA/pF, -1.89 pA/pF). (B-D) Na+/Ca2+ exchange current (INaCa), Ca2+ concentration in sarcoplasmic reticulum ([Ca2+]SR) and leakage current from SR to cytoplasm (Ileak) during the entire simulating period of 800 s with the current densities of (IK1, If) at (0.297 pA/pF, -2.52 pA/pF). (TIF) [file pcbi.1008177.s003.tif]

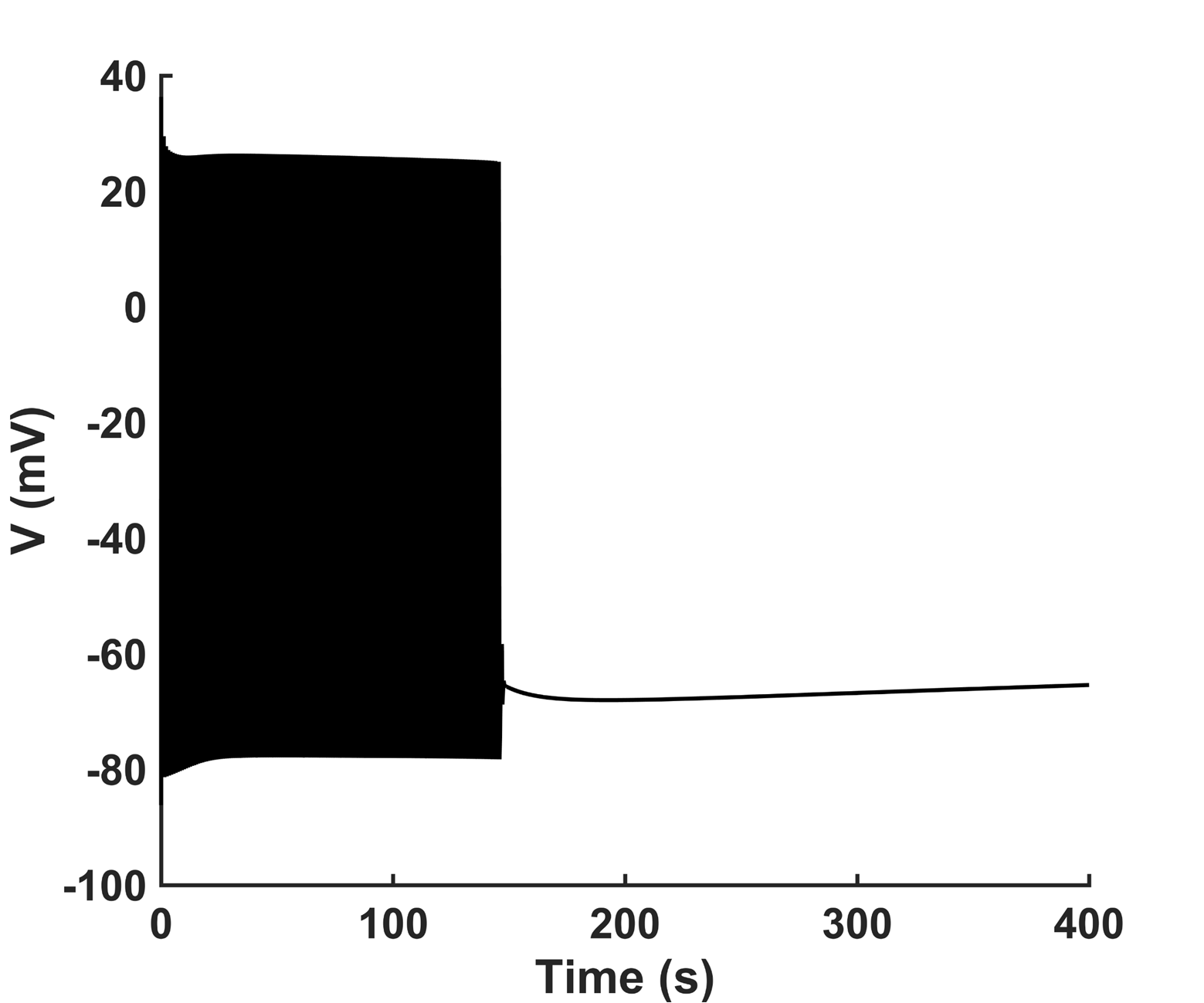

Supplement: S4 Fig — Membrane potential (V) during the entire simulation period of 400 s with the current densities of (IK1, If) at (0.178 pA/pF, -0.63 pA/pF). (TIF) [file pcbi.1008177.s004.tif]

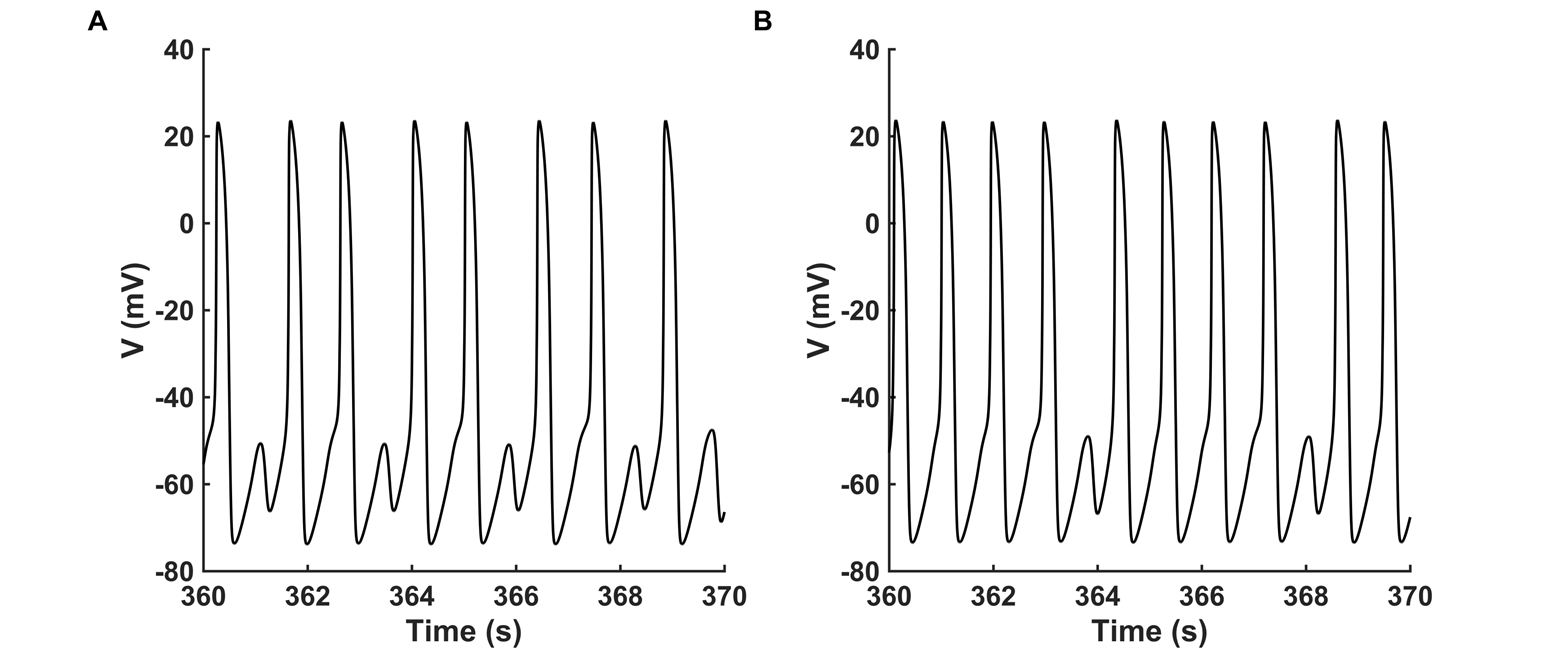

Supplement: S5 Fig — (A-B) Membrane potential (V) with the current densities of (IK1, If) at (0.297 pA/pF, -3.15 pA/pF) and (0.277 pA/pF, -3.15 pA/pF) during simulating time course of 360–370 s. (TIF) [file pcbi.1008177.s005.tif]

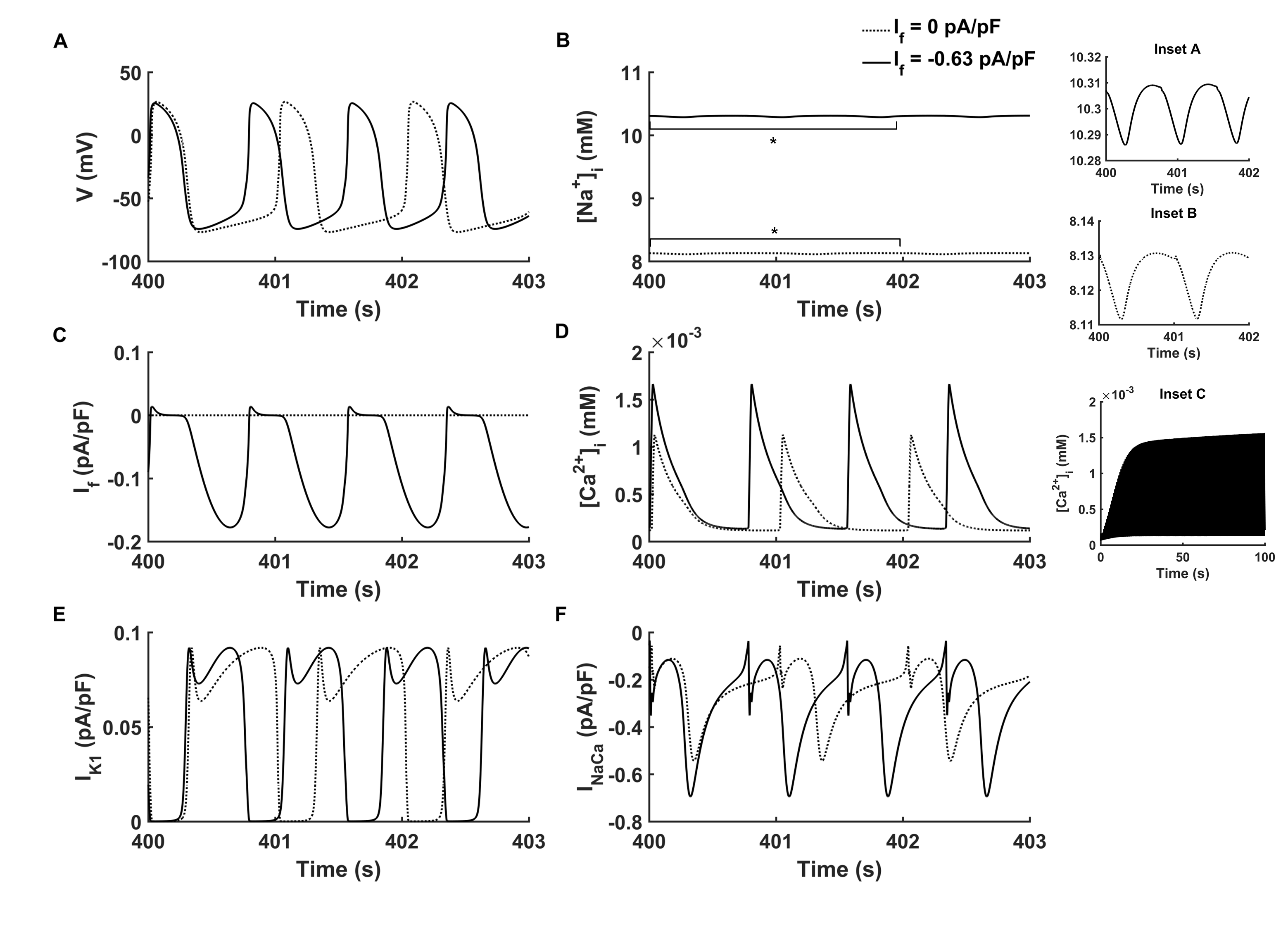

Supplement: S6 Fig — (A-F) The membrane potential (V), intracellular Na+ concentration ([Na+]i), “funny” current (If), intracellular Ca2+ concentration ([Ca2+]i), inward rectifier potassium channel current (IK1) and Na+/Ca2+ exchange current (INaCa) during simulating time course of 400–403 s when the current densities of (IK1, If) are at (0.05 pA/pF, 0 pA/pF) and (0.05 pA/pF, -0.63 pA/pF) (dotted and solid line respectively). (Inset A-B) Expanded plots of [Na+]i traces for the time course marked by the horizontal brackets with asterisks in (B). (Inset C) The change of [Ca2+]i with simulating time course of 0–100 s. (TIF) [file pcbi.1008177.s006.tif]

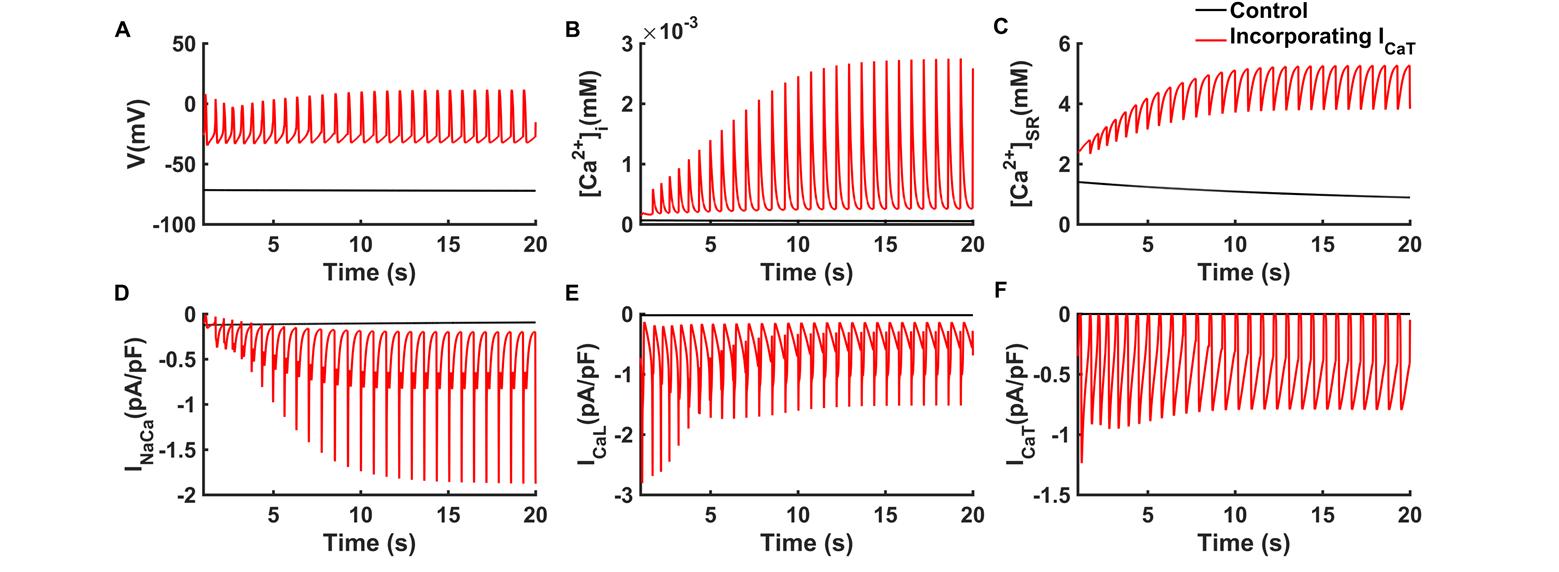

Supplement: S7 Fig — (A-F) Membrane potential (V), intracellular Ca2+ concentration ([Ca2+]i), Ca2+ concentration in sarcoplasmic reticulum ([Ca2+]SR), Na+/Ca2+ exchange current (INaCa), L-type calcium channel current (ICaL) and T-type calcium channel current (ICaT) with the current densities of (IK1, If) at (0.297 pA/pF, -0.63 pA/pF) during the simulating period of 0–20 s. (TIF) [file pcbi.1008177.s007.tif]

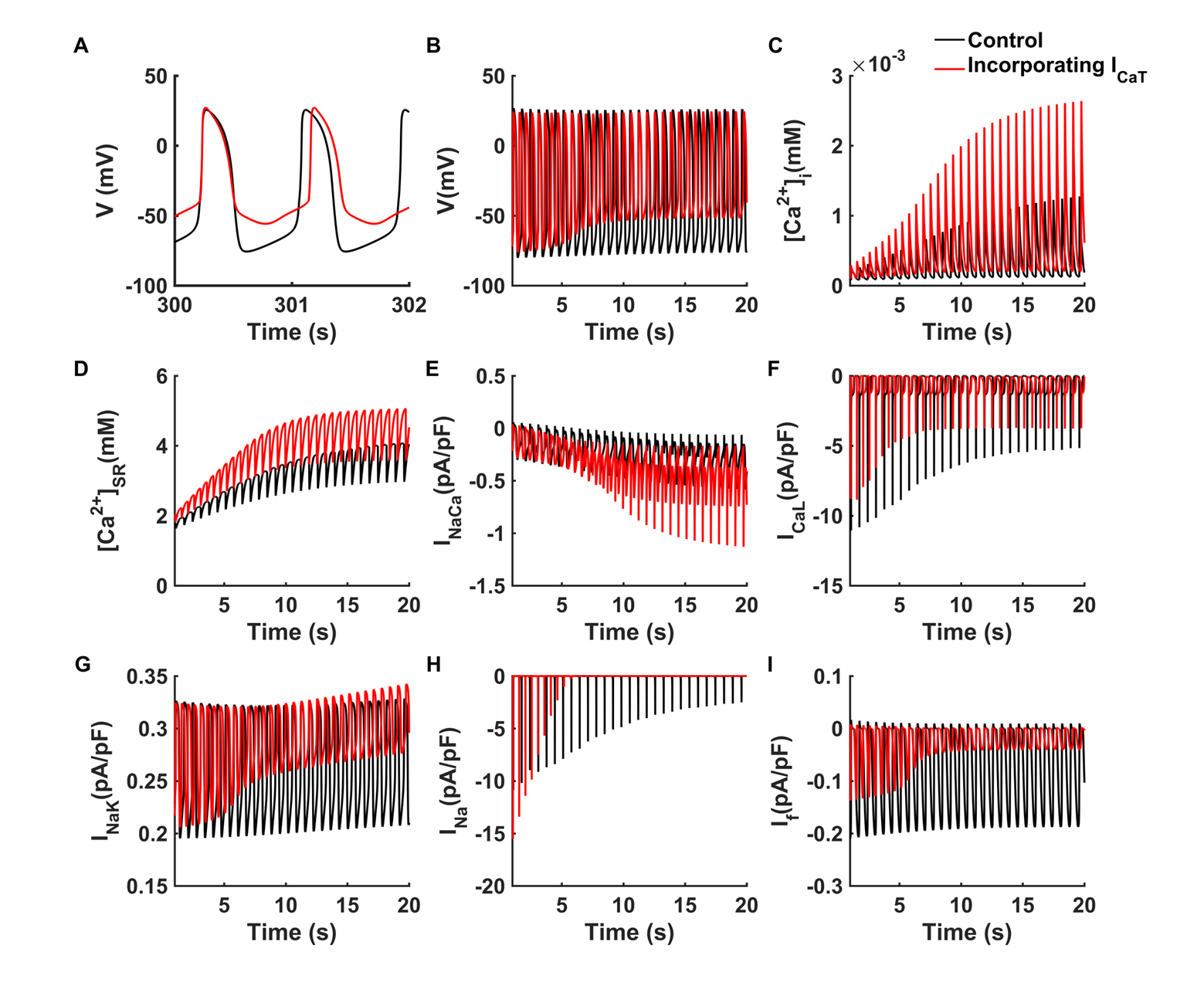

Supplement: S8 Fig — (A) Membrane potential (V) during the simulating period of 300–302 s. (B-H) Membrane potential (V), intracellular Ca2+ concentration ([Ca2+]i), Ca2+ concentration in sarcoplasmic reticulum ([Ca2+]SR), Na+/Ca2+ exchange current (INaCa), L-type calcium channel current (ICaL), Na+/K+ pumping current (INaK), fast sodium current (INa) and “funny” current (If) with the current densities of (IK1, If) at (0.099 pA/pF, -0.63 pA/pF) during the simulating period of 0–20 s. (TIF) [file pcbi.1008177.s008.tif]
