# Supplementary material for: Reciprocal interaction between IK1 and If in biological pacemakers: A simulation study
Source: PLoS Comput Biol. 2021 Mar 10;17(3):e1008177. doi: 10.1371/journal.pcbi.1008177 (PMC7984617; doi:10.1371/journal.pcbi.1008177)
Supplement: S1 Text — (DOC) [file pcbi.1008177.s009.doc]

**Prolonged cycle length at greater I_f_ density**

**
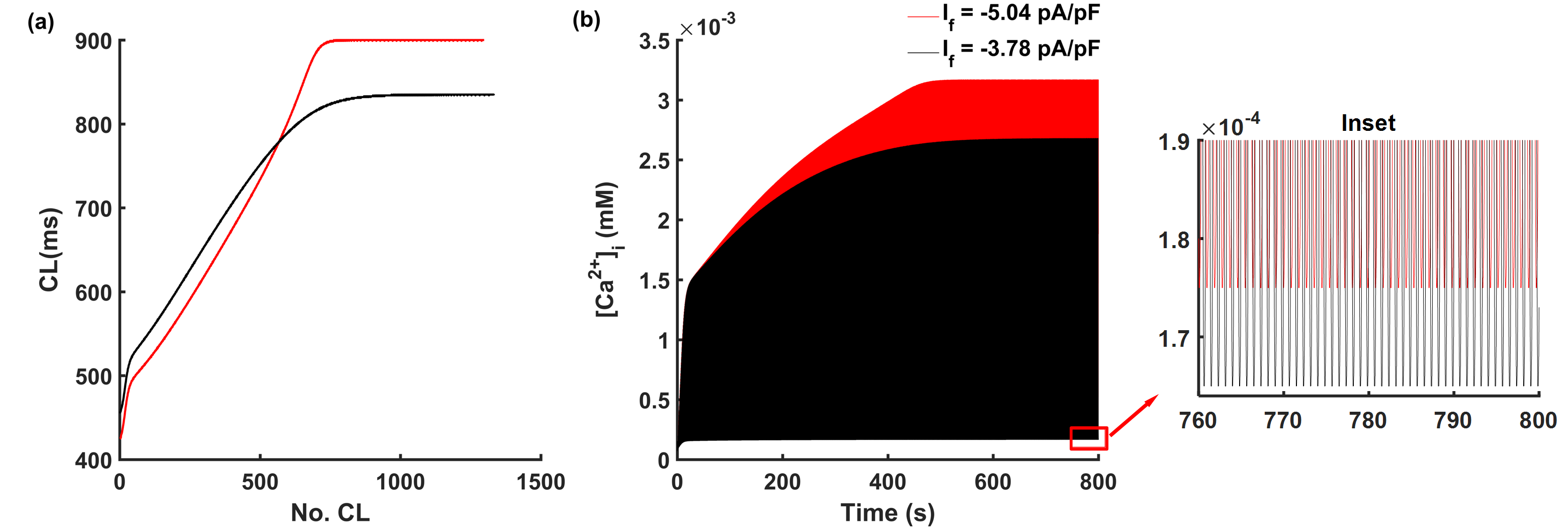
**

**Fig A.** The change of cycle length (a) and intracellular Ca^2+^ concentration ([Ca^2+^]_i_) (b) during the simulation period from 0 to 800 s. (Inset) Expanded plots of [Ca^2+^]_i_ traces for the time course marked by the red rectangle in (b).

**
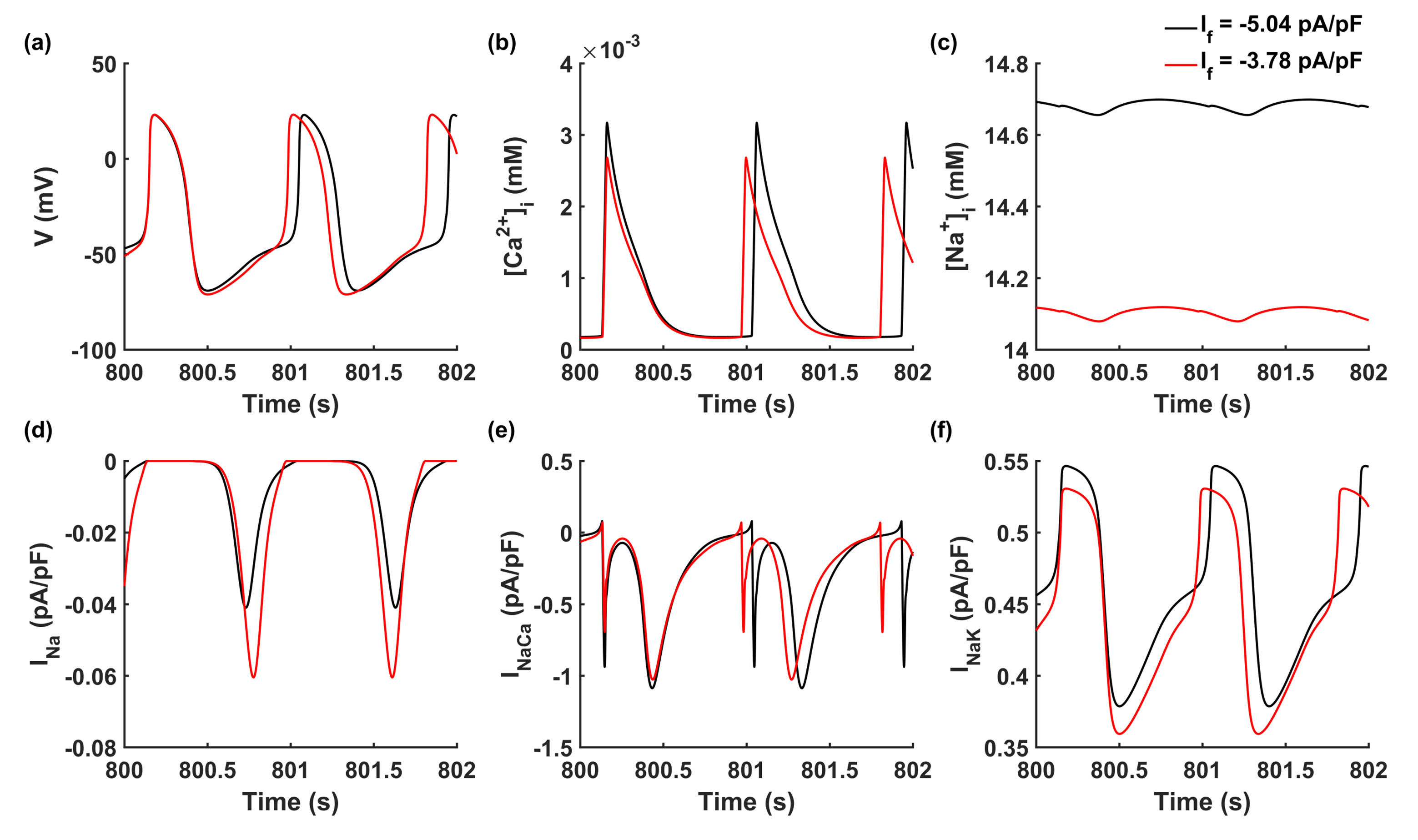
**

**Fig B.** (a-f) Membrane potential (V), intracellular Ca^2+^ concentration ([Ca^2+^]_i_), intracellular Na^+^ concentration ([Na^+^]_i_), fast sodium current (I_Na_), Na^+^/Ca^2+^ exchange current (I_NaCa_) and Na^+^/K^+^ pumping current (I_NaK_) with the current densities of (I_K1_, I_f_) at (0.198 pA/pF, -5.04 pA/pF) and (0.198 pA/pF, -3.78 pA/pF) (black and red lines respectively) during simulation period from 800 to 802 s.

**Table A Parameters of membrane potential in Fig 2 A.**

| I_f_ density (pA/pF) | Cycle length (ms) | Action potential duration (ms) | Diastolic interval  (ms) | Maximum diastolic potential (mV) |
| --- | --- | --- | --- | --- |
| -3.78 | 835 | 313 | 540 | -71 |
| -5.04 | 900 | 313 | 587 | -69 |


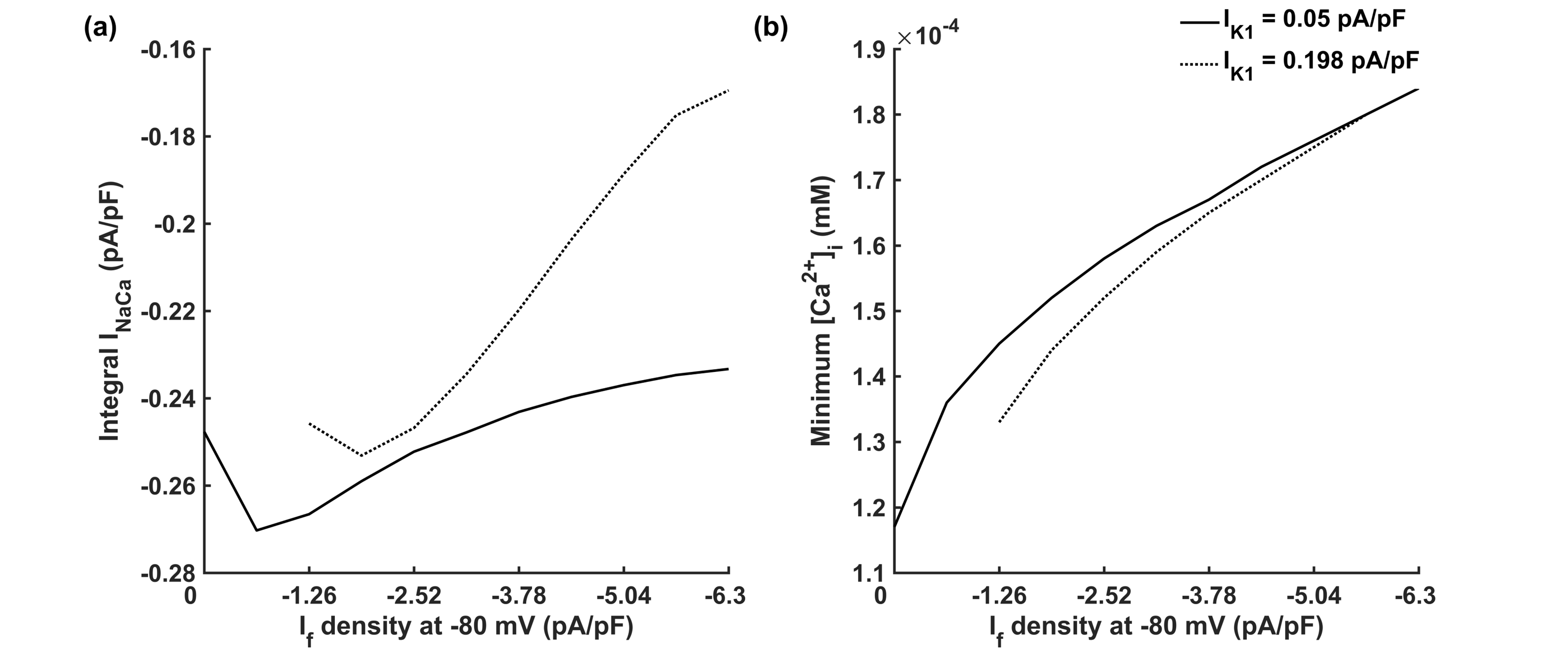


**Fig C.** (a-b) Change of the normalized total integral of Na^+^/Ca^2+^ exchange current during DI phase (Integral I_NaCa_) and minimum intracellular Ca^2+^ concentration (Minimum [Ca^2+^]_i_) with the increase of I_f_ from 0 to -6.3 pA/pF when I_K1_ density is 0.05 (solid line) and 0.198 pA/pF (dotted line).
