# Supplementary material for: Reciprocal interaction between IK1 and If in biological pacemakers: A simulation study
Source: PLoS Comput Biol. 2021 Mar 10;17(3):e1008177. doi: 10.1371/journal.pcbi.1008177 (PMC7984617; doi:10.1371/journal.pcbi.1008177)
Supplement: S2 Text — (DOC) [file pcbi.1008177.s010.doc]

**Model-dependence test**

We repeated the simulations of the biological pacemaker model based on a modified O’Hara-Rudy model of human ventricular cells by manipulating the inward rectifier potassium channel current (I_K1_) and “funny” current (I_f_). Simulation results showed that when I_K1_ was suppressed by 80%, the ventricular myocyte could not be transformed into the spontaneous pacemaker cell. Only I_f_ was incorporated (the current densities of (I_K1_, I_f_) were held at (0.198 pA/pF, -0.63 pA/pF) at -80 mV in I-V curve), can stable spontaneous pacemaking activity be induced with a computed cycle length (CL) of 850 ms, an action potential duration at 90% repolarization (APD_90_) of 358 ms and a maximum diastolic potential (MDP) of -85.1 mV (Fig A, solid line). A further decrease in I_K1_ density at 0.178 pA/pF produced a stronger pacemaker with a CL at 831 ms (Fig A, dotted line), which meant the inhabitation of I_K1_ could promote spontaneous action potentials. In addition, a further increase in I_f_ can also promote pacemaking activity (e.g., CL was 772 ms with (I_K1_, I_f_) at (0.198 pA/pF, -0.756 pA/pF) (Fig A, dashed line)). However, excessive I_f_ led to the termination of the pacemaking activity (e.g., (I_K1_, I_f_) at (0.198 pA/pF, -1.26 pA/pF), Fig B) because the MDP was too positive to achieve the activation potential of depolarizing currents.


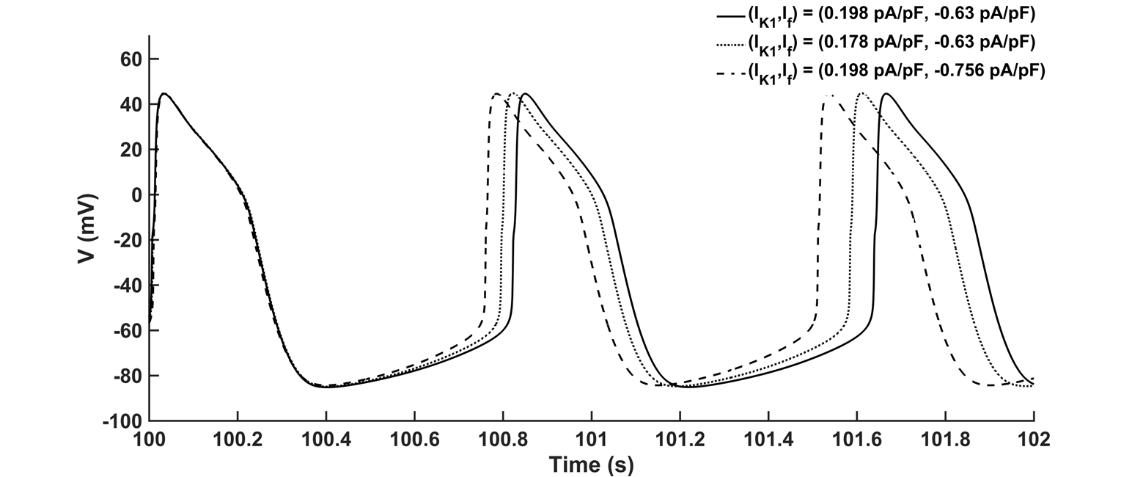


**Fig A. Stable pacemaking behaviour.**

Membrane potential (V) with the current densities of (I_K1_, I_f_) at (0.198 pA/pF, -0.63 pA/pF), (0.178 pA/pF, -0.63 pA/pF) and (0.198 pA/pF, -0.756 pA/pF) (solid, dotted and dashed line respectively) based on a human ventricular myocytes model.


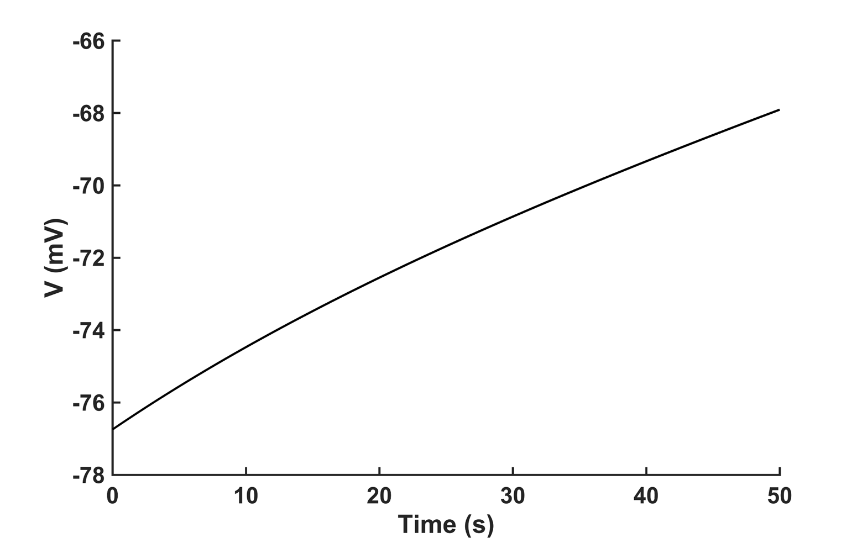


**Fig B. Failed pacemaking activity.**

Membrane potential (V) with the current densities of (I_K1_, I_f_) at (0.198 pA/pF, -1.26 pA/pF) based on a human ventricular myocytes model.

1. O'Hara T, Virag L, Varro A, Rudy Y. Simulation of the undiseased human cardiac ventricular action potential: model formulation and experimental validation. PLoS computational biology. 2011;7(5):e1002061.

2. Whittaker DG, Ni H, Benson AP, Hancox JC, Zhang H. Computational Analysis of the Mode of Action of Disopyramide and Quinidine on hERG-Linked Short QT Syndrome in Human Ventricles. Frontiers in physiology. 2017;8:759.

3. Fabbri A, Fantini M, Wilders R, Severi S. Computational analysis of the human sinus node action potential: model development and effects of mutations. The Journal of physiology. 2017;595(7):2365-96.
